# A Novel TNFSF-Based Signature Predicts the Prognosis and Immunosuppressive Status of Lower-Grade Glioma

**DOI:** 10.1155/2022/3194996

**Published:** 2022-05-09

**Authors:** Rui Tao, Qi Liu, Ruoyu Huang, Kuanyu Wang, Zhiyan Sun, Pei Yang, Jiangfei Wang

**Affiliations:** ^1^Department of Neurosurgery, Beijing Tiantan Hospital, Capital Medical University, Beijing, China; ^2^Department of Neurosurgery, Beijing Neurosurgical Institute, Capital Medical University, Beijing, China; ^3^Gamma Knife Center, Beijing Tiantan Hospital, Capital Medical University, Beijing, China

## Abstract

**Purpose:**

Tumour necrosis factor (TNF) superfamilies play important roles in cell proliferation, migration, differentiation, and apoptosis. We believe that TNF has a huge potential and might cast new insight into antitumour therapies. Therefore, we established this signature based on TNF superfamilies.

**Results:**

A six-gene signature derived from the TNF superfamilies was established. The Riskscore correlated significantly with the expression of immune checkpoint genes and infiltrating M2 macrophages in the tumour specimen. This signature was also associated with mutations in genes that regulate tumour cell proliferation. Univariate and multivariate regression analyses further confirmed the Riskscore, TNFRSF11b, and TNFRSF12a as independent risk factors in The Cancer Genome Atlas and Chinese Glioma Genome Atlas datasets.

**Conclusion:**

Our signature could accurately predict the prognosis of lower-grade gliomas (LGG). In addition, this six-gene signature could predict the immunosuppressive status of LGG and provide evidence that TNF superfamilies had correlations with some critical mutations that could be effectively targeted now.

## 1. Introduction

Gliomas are lethal malignant neoplasms of the brain and other parts of the central nervous systems. In the past, low-grade gliomas were primarily categorised as World Health Organization (WHO) grade II diffuse gliomas, whereas high-grade gliomas were classified as WHO grade III/IV gliomas. Nevertheless, this histology-based classification has been gradually replaced by the novel concept of lower-grade gliomas (LGGs) consisting of WHO grade II and III diffuse gliomas. In the United States, the annual age-adjusted incidence rates of diffuse astrocytoma, anaplastic astrocytoma, and oligodendroglioma were 0.45/100000, 0.42/100000, and 0.23/100000, respectively. The median survival associated with these three types of gliomas were 36, 18, and 119 months, respectively, and the corresponding five-year survival rates were 43.1%, 22.7%, and 69.6%, respectively [1–3].

In recent years, the previously identified molecule tumour necrosis factor (TNF) has received renewed attention. This molecule was first found to have a tumour-inhibiting effect in patients with sarcoma infected with bacteria. The TNF family refers to a set of proteins consisting of 29 receptors and 19 ligands. TNF and TNF receptor (TNFR) superfamilies (TNFSF/TNFRSF) are believed to play important roles in cell proliferation, migration, differentiation, survival, and apoptosis [4]. TNF inhibitors can synergise with epidermal growth factor receptor (EGFR) or immune checkpoint inhibitors to enhance their antitumour capacities. Consequently, TNF superfamilies are regarded as promising targets that can be integrated into current therapeutic strategies [5–7]. Currently, several costimulatory receptor agonistic antibodies targeting 4-IBB (TNFRSF9 or CD137) and OX40 (TNFRSF4) have undergone phase I trials (ClinicalTrials.gov Identifier: NCT02179918; ClinicalTrials.gov Identifier: NCT02274155) [8–10].

The phenotypes of TNF superfamilies in lung cancer have been reported to be strongly related with patient prognosis [11]. The phenotype of TNF superfamily members refers to the expression levels of select genes of this superfamily. This specific gene expression profile could predict the prognosis of some types of malignancies and, accordingly, be deemed as TNF-based phenotypes. We hypothesised that a similar relationship may exist in LGGs. Considering the major potential of TNF superfamilies to provide new insights into antitumour therapies, we established this signature based on TNFSF/TNFRSF.

## 2. Materials and Methods

### 2.1. Clinical Information and RNA Expression Data

We selected 443, 449, and 108 patients with effective clinical and follow-up information along with RNA expression data from the Chinese Glioma Genome Atlas (CGGA), The Cancer Genome Atlas (TCGA), and the GSE16011 dataset, respectively. We also obtained access to data from the REMBRANDT cohort, although the clinical information for this cohort was incomplete. Of note, the CGGA database provides high-quality functional genomic data resources for Chinese cases of glioma and facilitated our research immensely [12].

All patients selected in this study were diagnosed with WHO grade II/III gliomas based on histological diagnostic criteria. Demographic and clinical statistics from the aforementioned database are shown in Table 1. Specific data were downloaded from the official website of TCGA (https://cancergenome.nih.gov/), CGGA (http://www.cgga.org.cn/), GSE16011 (https://www.ncbi.nlm.nih.gov/), and REMBRANDT (http://www.betastasis.com/glioma/rembrandt/). The RNA-seq data were all log_2_-transformed and normalised before our analysis procedure. A gene list of members belonging to the TNF superfamily retrieved from a previously published review was applied to our study to identify the six-gene signature [4]. This research was approved by the ethics committee of Tiantan Hospital, affiliated with Capital Medical University.

### 2.2. Identification of TNFSF/TNFRSF Superfamily Signature

A total of 48 genes encoding either TNF ligands or receptors with available gene expression information in the previously mentioned database were used in our study. We first established a LASSO Cox model to predict prognostic effectiveness using the optimal lambda [13]. TCGA clinical and RNA-seq data were used as the training set, while the CGGA, GSE16011, and REMBRANDT data were utilised for validation.

### 2.3. Statistical Analysis

SPSS version 25.0 and the R project were used for statistical analyses. Overall survival was the main prognostic indicator. It was defined as the interval between the first diagnosis of grade II/III gliomas and the death or last follow-up of the patient. We calculated the Riskscore by employing the LASSO Cox model. Kaplan–Meier curves and log-rank tests were then used to compare survival outcomes between the high-risk and low-risk groups. The median Riskscore was designated as the cut-off value that discriminated between the high-risk and low-risk groups. Patients with Riskscore higher and lower than the cut-off value were classified into the high-risk and low-risk groups, respectively. We took advantage of the R project to draw the receiver operating characteristic curves (ROC) and determine the area under the curve, which examined the prognostic effectiveness of this six-gene signature [14]. Box-and-scatter figures were plotted with the help of R package “ggplot2.” The chi-square test, univariate Cox regression, and multivariate Cox regression analyses were performed using SPSS 25.0. *p* ≤ 0.05 was considered statistically significant. Gene set enrichment analysis (GSEA) was performed with GSEA 4.1.0., while Gene Ontology (GO) analysis was performed online (https://david.ncifcrf.gov/). The common pathways or processes of GSEA and GO analyses were singled out to plot the heat map with the aid of R package “ComplexHeatmap” [15].

To further investigate whether the selected genes in our signature influenced the tumour microenvironment and led to immune evasion in LGGs, we calculated the constitution of immune cell infiltration in each tumour sample with the aid of CIBERSORT and LM22 signatures (http://cibersort.stanford.edu/) [16, 17]. After analysing the discrepancies of infiltrated immune cells within tumours between the high-risk and low-risk groups, the expression of immune checkpoints was shown with the R package “ggpubr.” The mutated profiles of TCGA were obtained from The Cancer Immunome Atlas (https://tcia.at/home) and compared between high-risk and low-risk groups. The landscape of gene mutation statuses and the pathway alterations among LGG samples in TCGA dataset was demonstrated with the help of the R package “maftools.”

## 3. Results

### 3.1. Demographic Statistics and Clinical Features of the Grade II/III Patient Cohort

A total of 449 patients with low-grade gliomas (LGG, WHO II/III gliomas), which consisted of 251 men and 198 women, were selected from TCGA database. The corresponding numbers of patients from the CGGA database were 251 and 192, respectively. In TCGA, CGGA, and GSE16011, patients younger than 45 years old were found more likely to develop LGGs (WHO II/III gliomas). The number of patients with grade II and grade III gliomas was 213 and 236, 188 and 255, 23 and 85, and 72 and 71 in TCGA, CGGA, GSE16011, and REMBRANDT cohorts, respectively. Isocitrate dehydrogenase (IDH) mutation status, 1p/19q codeletion status, and grade were also introduced in our study for further analysis of the signature. The median overall survival (OS) and range of OS values were 87.394 months and 0.033–211.027 months in TCGA cohort, 83.700 months and 1.7–167.6 months in the CGGA cohort, 41.640 months and 0.24–248.16 months in the GSE16011 cohort, and 42.60 months and 0.2–251.733 months in the REMBRANDT cohort. The 1-, 2-, and 5-year survival rates in TCGA, CGGA, GSE16011, and REMBRANDT cohorts are shown in Table 1, along with other detailed information.

### 3.2. Six-Gene Signature Establishment

Other than the widely known pro-inflammatory properties, the antitumour responses of this superfamily have not been fully understood. We propose that the expression of TNFSF/TNFRSF and the survival outcomes of grade II/III patients are connected. To verify this interaction, we used the LASSO Cox model to establish a signature that could predict prognosis. Using this model, we confirmed a six-gene signature (Supplementary Table 1). We used the product of each patient's gene expression and the coefficient from the LASSO Cox model as the Riskscore, and the formula is as follows:

(1) $$\begin{array}{c} \text{Riskscore}=\left(0.128514092429542\times \text{CD}70\right)+\left(-0.061112802949453\times \text{EDA}\right)+\left(0.463397817144414\times \text{TNFRSF}11\text{B}\right)+\left(0.279587869990881\times \text{TNFRSF}12\text{A}\right)+\left(0.0154761687424339\times \text{TNFRSF}14\right)+\left(-0.029856055076389\times \text{TNFRSF}25\right). \end{array}$$

**Figure 1:**
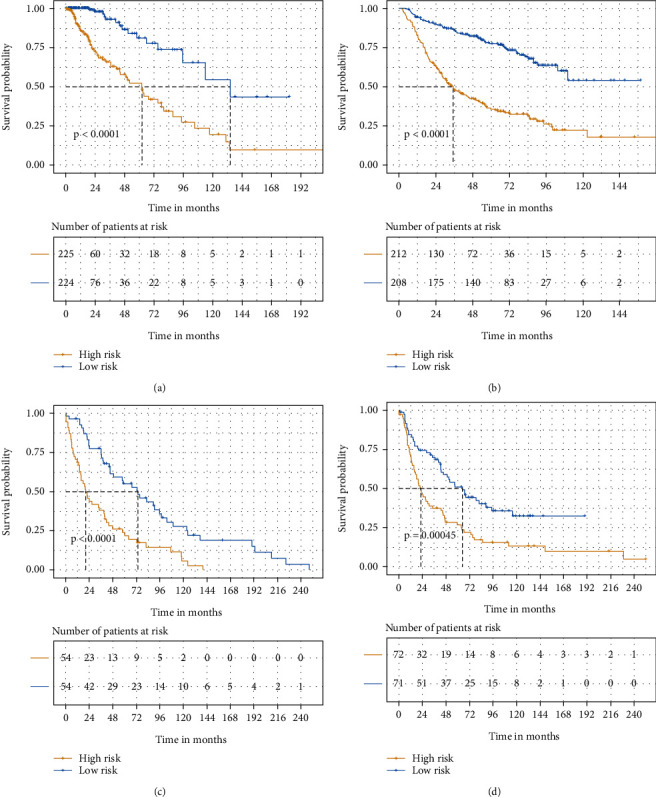
Kaplan–Meier survival curve showing overall survival of lower-grade gliomas (LGGs) in high-risk and low-risk groups. (a) Kaplan–Meier survival curve of the TCGA cohort. (b) Kaplan–Meier survival curve of the CGGA cohort. (c) Kaplan–Meier survival curve of the GSE16011 cohort. (d) Kaplan–Meier survival curve of the REMBRANDT cohort.

**Figure 2:**
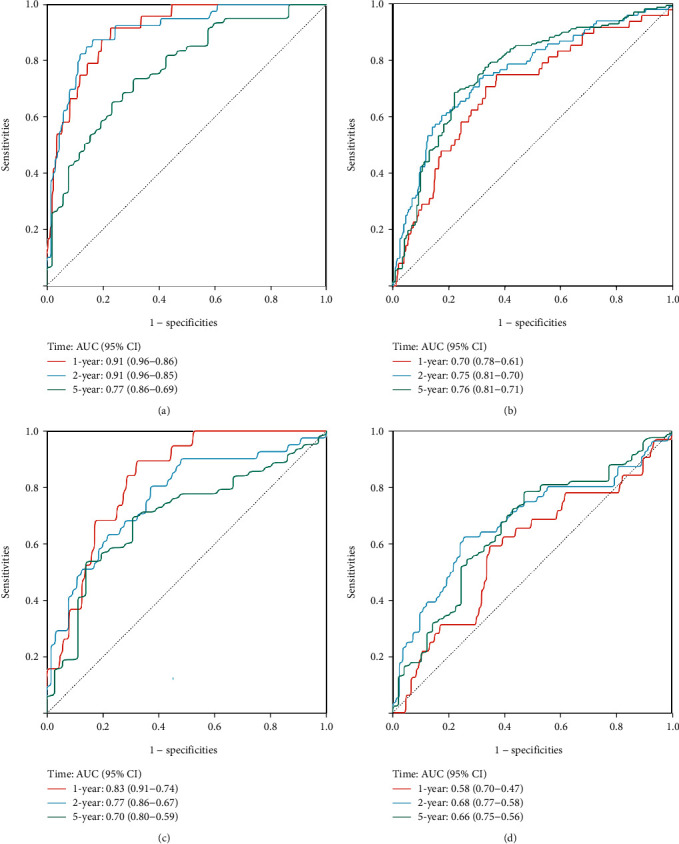
Time-dependent receiver operating characteristic curve of six-gene signature in the TCGA (a), CGGA (b), GSE16011 (c), and REMBRANDT (d) database.

Patients were divided into high- and low-risk group according to the median Riskscore (cut-off value) in these public databases. The number of patients in the low- and high-risk groups was 224 and 225, 221 and 222, 54 and 54, and 71 and 72 in TCGA, CGGA, GSE16011, and REMBRANDT cohorts, respectively. We then performed Kaplan–Meier survival analysis in TCGA cohort, and patients in the high-risk group had worse prognostic outcomes than those in the low-risk group (*p* < 0.0001). The CGGA, GSE16011, and REMBRANDT cohorts were used as validation datasets. Similarly, patients in the low-risk group had better prognoses than those in the high-risk group in the validation cohorts. Therefore, patients with higher Riskscore were at a higher risk and thus had poorer survival outcomes. We could intuitively observe the differences in survival outcomes between the low- and high-risk groups determined by the cut-off value through the Kaplan–Meier curve displayed (Figures 1(a)–1(d)). ROC curves and nomograms were plotted to examine the predictive effectiveness of this signature (Figures 2(a)–2(d); Supplementary Figure 1a, 1b, c, d).

### 3.3. Analysis of the Risk Factors of LGG Patients

To analyse the risk factors that affected the OS of LGG patients, we performed univariate and multivariate Cox regression analyses in TCGA, CGGA, and GSE16011 datasets. First, we conducted a univariate analysis and found that age, IDH mutation status, 1p/19q codeletion status, MGMT promoter status, grade, and Riskscore had significant correlations with OS in TCGA cohort (Supplementary Table 2). All these factors with the exception of age and MGMT promoter status were also significantly associated with OS in the CGGA cohort (Supplementary Table 3). In the GSE16011 cohort, age, EGFR status, 1p/19q codeletion status, Karnofsky performance score, and Riskscore were clearly related to OS (Supplementary Table 3).

**Figure 3:**
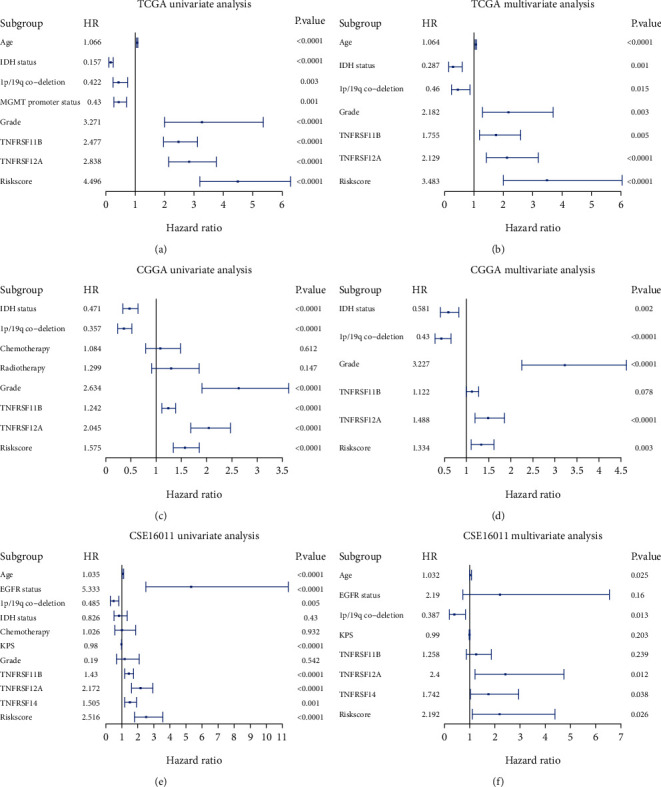
These forest maps intuitively showed the protective or risk factors of univariate and multivariate Cox analysis in the TCGA, CGGA, and GSE16011 cohort. (a) Univariate Cox analysis in the TCGA cohort. (b) Multivariate Cox analysis in the TCGA cohort. (c) Univariate Cox analysis in the CGGA cohort. (d) Multivariate Cox analysis in the CGGA cohort. (e) Univariate Cox analysis in the GSE16011 cohort. (f) Multivariate Cox analysis in the GSE16011 cohort.

We next added these univariates into a multivariate regression analysis program and found that age, IDH mutation status, 1p/19q codeletion status, grade, and Riskscore significantly affected the OS of grade II/III patients in TCGA dataset (Supplementary Table 2). IDH mutation status, 1p/19q codeletion status, grade, and Riskscore significantly influenced survival outcomes in the CGGA dataset (Supplementary Table 3), and age, 1p/19q codeletion status, and Riskscore significantly influenced survival outcomes in the GSE16011 dataset (Supplementary Table 4). The Riskscore was an independent risk factor for OS in TCGA, CGGA, and GSE16011 cohorts. In addition, we verified that TNFRSF12A significantly influenced the OS of patients through multivariate Cox regression analysis in TCGA and CGGA cohorts (Figure 3, Supplementary Tables 2, 3, and 4).

### 3.4. Exploration of the Biological Processes and Pathways That Correlated with the Riskscore

**Figure 4:**
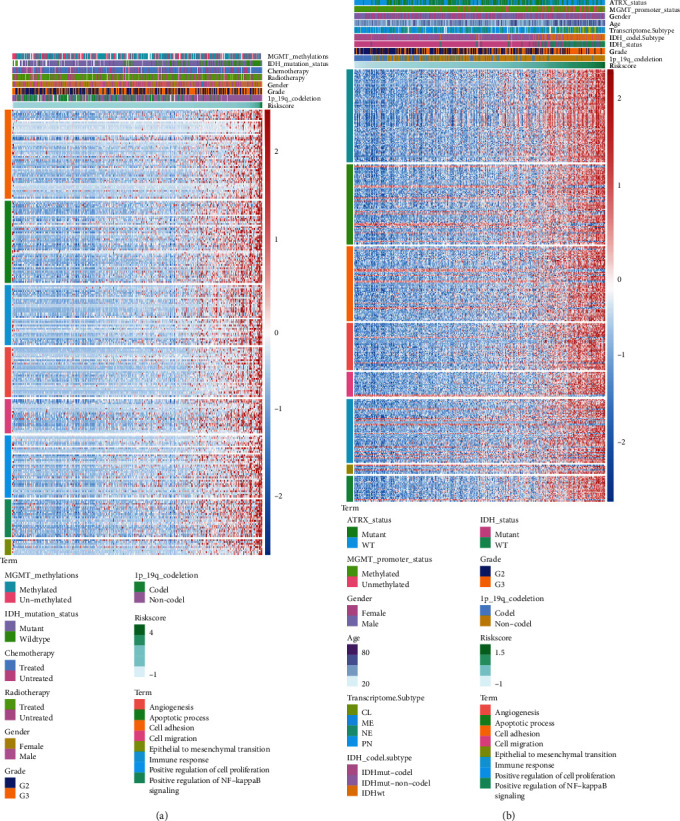
Gene Ontology analysis demonstrating the biological processes related to Riskscore in the TCGA (a) and CGGA (b) databases.

Since the Riskscore was identified as an independent risk factor through multivariate Cox regression analysis, we needed to further determine the underlying biological processes and pathways. First, two gene sets from TCGA and CGGA RNA expression datasets that separately had linear correlations with the Riskscore were selected for GO analysis. The correlated genes are listed in the supplementary materials. Identical processes and pathways in TCGA and CGGA databases were chosen: “apoptotic process,” “angiogenesis,” “epithelial to mesenchymal transition,” “positive regulation of I-*κ*B kinase/NF-*κ*B signalling,” “positive regulation of cell proliferation,” “immune response,” “cell adhesion,” and “cell migration.” Heatmaps of TCGA and CGGA datasets were also plotted, and they clearly showed that as the Riskscore increased, the expression of the genes involved in the aforementioned pathways increased as well (Figures 4(a) and 4(b)). The results of the GO analysis in the GSE16011 and REMBRANDT are shown in the supplementary materials (Supplementary Figure 2a, 2b). We also performed GSEA in the high- and low-Riskscore groups on the basis of the cut-off value referring to the median Riskscore. GSEA demonstrated that the common pathways linked with a high Riskscore in both TCGA and CGGA cohorts were “angiogenesis,” “apoptosis,” “epithelial-mesenchymal transition,” “inflammatory response,” “P53 pathway,” “PI3K AKT MTOR signalling pathway,” “KRAS signalling up pathway,” and “TNF-*α* signalling via NF-*κ*B” (Supplementary Figure 3a, 3b). The results of GSEA of the GSE16011 dataset and REMBRANDT databases are displayed in supplementary materials as well (Supplementary Figure 3c, 3d).

**Figure 5:**
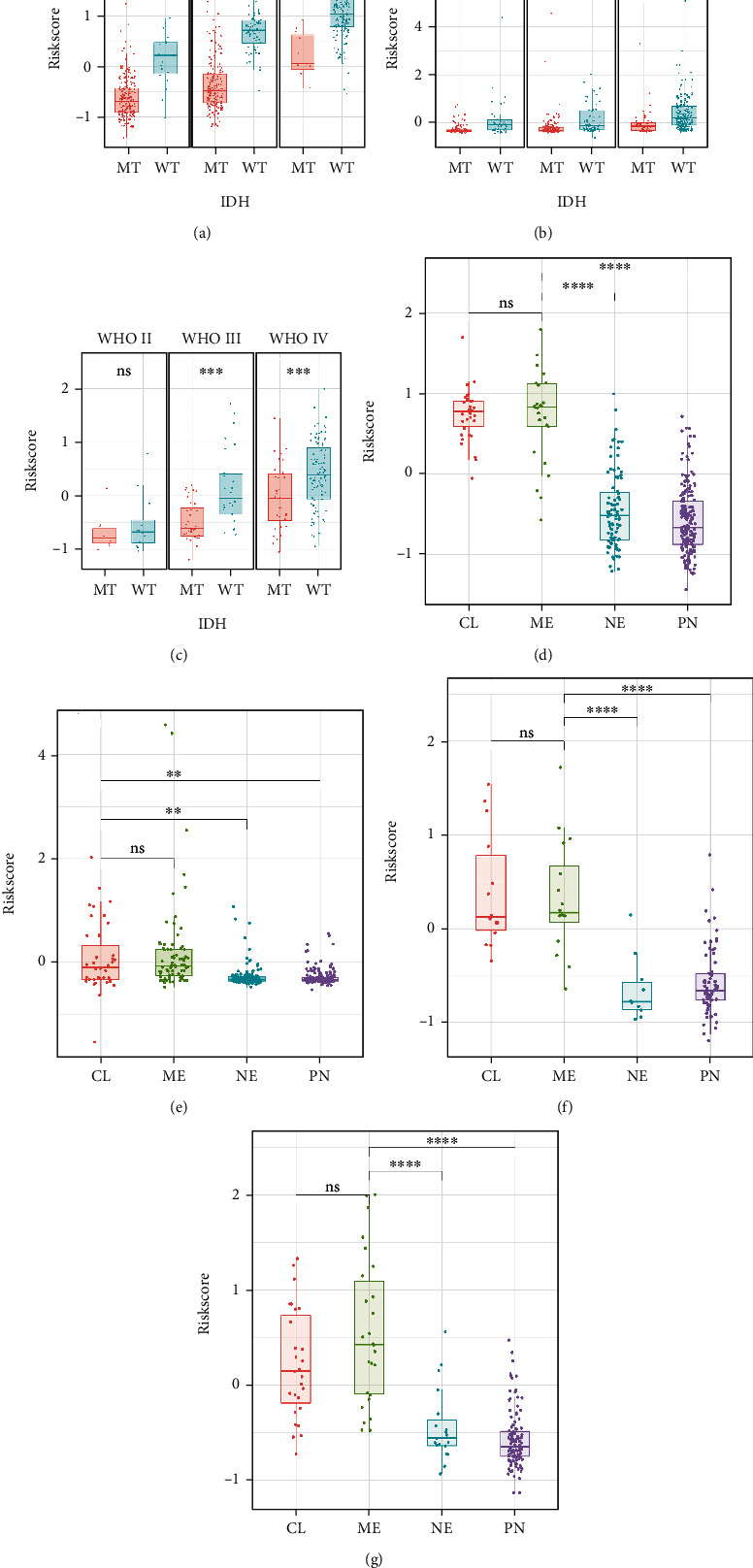
Distribution of Riskscore in all grades of gliomas according to IDH mutation status (a)–(c) and TCGA subtypes (d)–(g). (a) Riskscore distribution status in the TCGA database. (b) Riskscore distribution in the CGGA database. (c) Riskscore distribution status in the GSE16011 dataset. (d) Riskscore distribution status of the TCGA cohort. (e) Riskscore distribution status of the CGGA cohort. (f) Riskscore distribution status of the GSE16011 cohort. (g) Riskscore distribution status of the REMBRANDT cohort (MT: mutated; WT: wild type; CL: classical; ME: mesenchymal; NE: neural; PN: proneural; ∗: *p* < 0.05; ∗∗: *p* < 0.01; ∗∗∗: *p* < 0.001; ∗∗∗∗: *p* < 0.0001; ns: not significant).

Patients with gliomas of all grades were divided into IDH-wildtype and IDH-mutant groups and tested in TCGA, CGGA, and GSE16011 cohorts. We found that patients without IDH mutations had higher Riskscore within TCGA, CGGA, and GSE16011 cohorts (Figures 5(a)–5(c)). Similar analyses were conducted within a subset of grade II/III gliomas. The patients in TCGA, CGGA, and GSE16011 databases were distributed into four groups based on the transcriptome subtype: classical, mesenchymal, neural, and proneural. Those categorised in the classical and mesenchymal subtypes had a significantly higher Riskscore than those in the other two subtypes (Figures 5(d)–5(g)).

### 3.5. Immune Cell Infiltration and Immune Checkpoint Expression

**Figure 6:**
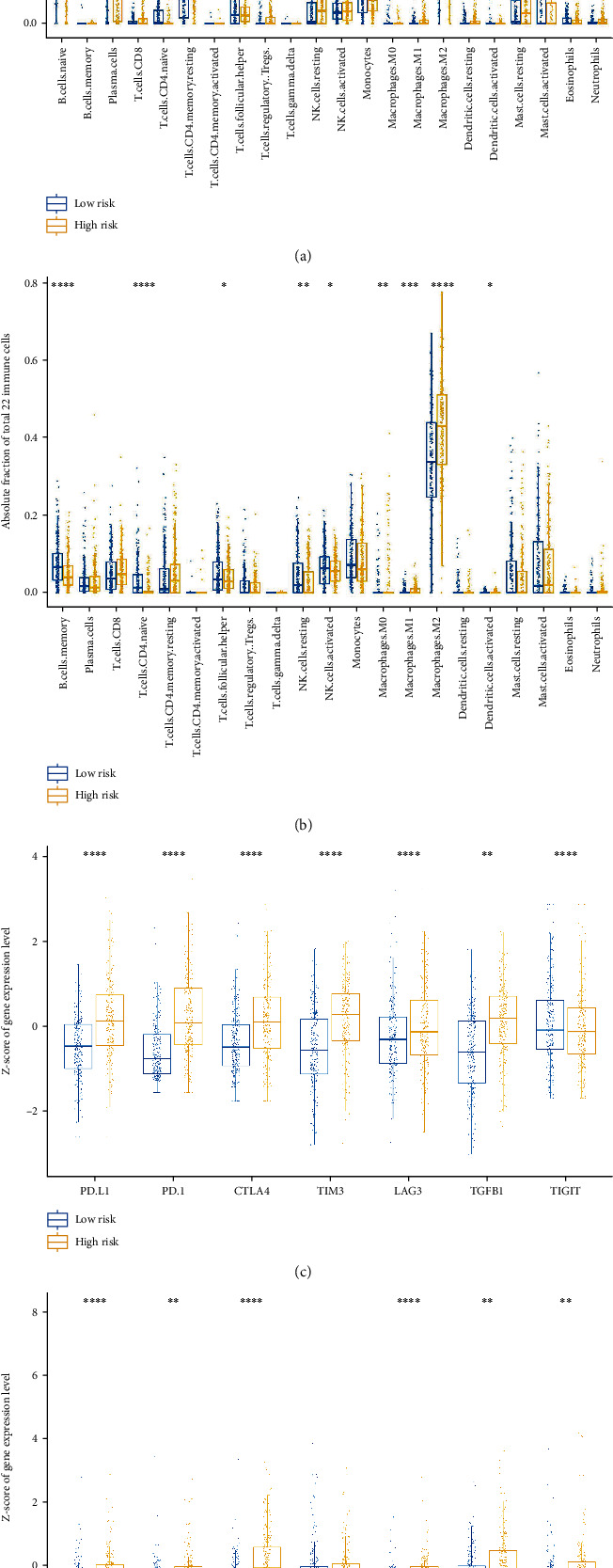
Distribution of tumour-infiltrating immune cells and immune checkpoint expression in high-risk and low-risk groups in LGGs. (a) Distribution of tumour-infiltrating immune cells in the TCGA cohort. (b) Distribution of tumour-infiltrating immune cells in the CGGA cohort. (c) Immune checkpoint molecule expression status in the TCGA database. (d) Immune checkpoint molecule expression status in the CGGA database. The fraction in (a) and (b) refers to the proportion of this kind of immune cell in all 22 types of immune cells calculated by CIBERSORT in one sample. Blue dots refer to immune cell fraction in the low-risk group and yellow dots the high-risk group. The expression in (c) and (d) indicates that the mRNA expression level of the selected immune checkpoints in the form of a *Z*-score. Blue dots refer to the expression of immune checkpoint molecules in the low-risk group and yellow dots in the high-risk group. ∗*p* < 0.05; ∗∗*p* < 0.01; ∗∗∗*p* < 0.001; ∗∗∗∗*p* < 0.0001.

To investigate whether there was a connection between our signature and immune response procedures, we performed CIBERSORT with LM22 to determine the proportion of 22 different immune cells in each sample from TCGA and CGGA databases. Notably, more M2-macrophages were found in the high-risk group than in the low-risk group (Figures 6(a) and 6(b)).

Since members of the TNF superfamilies are known to be correlated with the expression of immune checkpoint molecules, we performed a linear correlation analysis between Riskscore and the expression of six canonical biomarkers, including PD-1, PD-L1, CTLA4, TIM3, LAG3, and TGFB1, in TCGA and CGGA cohorts separately. We found that all these markers showed linear correlations with the Riskscore in TCGA cohort, as did PD-1, PD-L1, CTLA4, TIM-3, and TGFB1 in the CGGA database (Supplementary Table 5, 6). Later, we performed a comparison of these biomarkers between the high-risk and low-risk groups in TCGA and CGGA datasets. In both TCGA and CGGA cohorts, we found that the total expression of these six genes in the high-risk group was higher than that in the low-risk group (Figures 6(c) and 6(d)).

### 3.6. Mutated Profile of the High- and Low-Risk Groups in TCGA Dataset

**Figure 7:**
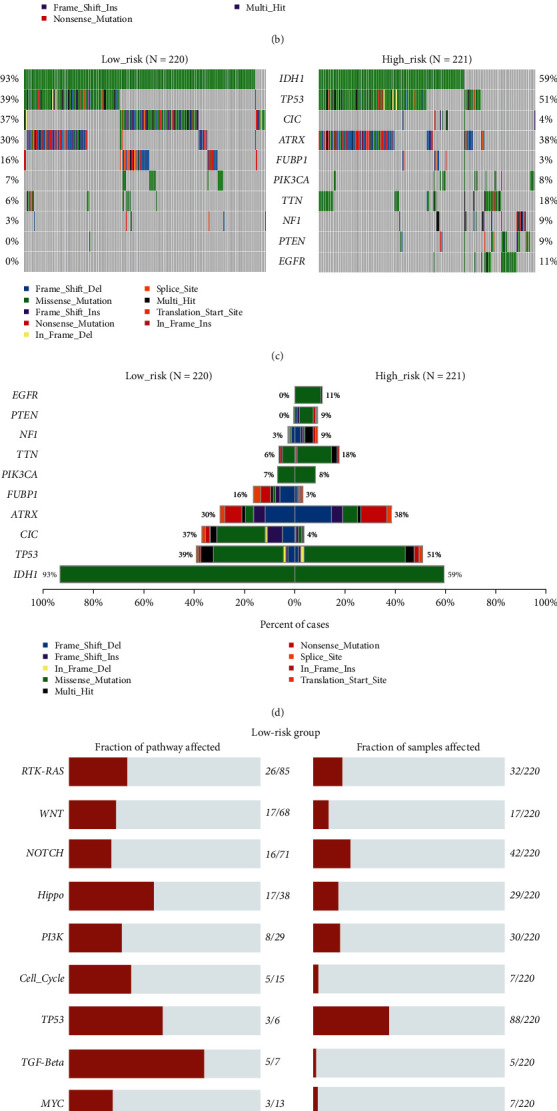
(a) Top 10 mutated genes with the highest frequency in the low-risk group in the TCGA database. (b) Top 10 mutated genes with the highest frequency in the high-risk group in the TCGA database. (c) These 10 genes are picked out which are either important or have a significant difference between the high-risk and low-risk group according to (a) and (b). (d) Bar plot intuitionally reflected the differences of these aforementioned 10 genes in (c) between the high-risk and low-risk group. Mutated genes were also demonstrated in (a)–(d). (e) Total genes mutated in the corresponding pathway composed of a settled gene list (left) and numbers of samples harbouring this specific altered pathway (right), in the low-risk group. (f) Similar to (e), it reflected the gene-mutated and pathway-altered pattern in the high-risk group (NFR2 pathway change was found exclusively in the high-risk group).

The top 10 types of mutated genes and related pathways were different in the high-risk and low-risk groups (Figures 7(a) and 7(b)). The frequencies of these mutations in the corresponding groups varied as well. The occurrence of IDH1 mutation in the low-risk group versus high-risk group was 93% vs. 59% (*p* < 0.0001); TP53, 39% vs. 51% (*p* = 0.011); CIC, 37% vs. 4% (*p* < 0.0001); ATRX, 30% vs. 38% (*p* = 0.076); FUBP1, 16% vs. 3% (*p* < 0.0001); EGFR, 0% vs. 11% (*p* < 0.0001); PTEN, 0% vs. 9% (*p* < 0.0001); TTN, 6% vs. 18% (*p* < 0.0001); PIK3CA, 7% vs. 8% (*p* = 0.597); and NF-1, 3% vs. 9% (*p* = 0.010) (Figures 7(c) and 7(d)). The fraction of samples affected by the RTK-RAS pathway alterations was significantly different between the low-risk and high-risk groups. Meanwhile, the PI3K and TP53 pathway showed a tendency of having a higher activated status in the high-risk group (RTK-RAS: 14.5% vs. 36.7%, *p* < 0.0001; PI3K: 13.6% vs. 24.4%, *p* = 0.004; TP53: 40% vs. 51.6%, *p* = 0.015, Figures 7(e) and 7(f)).

## 4. Discussion

Gliomas are recalcitrant malignant neoplasms. Even patients who underwent maximum-safe resections followed by high-dose chemotherapy and radiotherapy do not show significant prolongation of the OS and PFS. The tumours and the surrounding immunosuppressive microenvironment collectively led to drug resistance, tumour progression, and recurrence of gliomas. The appearance of an exhausted phenotype of cytotoxic T-lymphocytes (CTLs), recruitment of tumour-associated macrophages, and myeloid-derived suppressor cells are critical characteristics of an immunosuppressive microenvironment. One of the most important mechanisms underlying these findings is the overexpression of immune checkpoints. Some literatures found that immune checkpoint molecules correlated to the prognosis of gliomas [18, 19]. However, according to a phase III CheckMate 143 trial (ClinicalTrials.gov Identifier: NCT02017717), the anti­PD­1 antibody nivolumab did not show promising effects on prolonging the OS of patients with recurrent glioblastoma [20]. Duan et al. reported that the paucity of CD30L (TNFRSF8) expression could upregulate the expression of PD-1 on CD8+ T cells, resulting in the progression of gliomas [7]. This indicated that TNF might be correlated with immune checkpoints and, in turn, influenced the curative effect of immune checkpoint inhibitors. In addition, TNF has been hypothesised to enhance immunoreactivity as a costimulated receptor and overcome the limited application of immune checkpoint inhibitors because of peripheral tolerance and immunosuppression. Immune checkpoint inhibitors can activate nonspecific T cells and cause autoimmune responses. Jiang et al. reported that oncolytic adenovirus combined with the immune costimulator OX40 ligand (OX40L, TNFSF4) could enable immune cells to accurately recognise tumour-associated antigens and reduce the adverse effects caused by the activation of irrelevant T cells [21].

Because of the antitumour therapy potential of TNFs, we took advantage of the LASSO model consisting of six genes (*CD70*, *EDA*, *TNFRSF11B*, *TNFRSF12A*, *TNFRSF14*, and *TNFRSF25*); we constructed this signature filtered out from TNFSF/TNFRSF. CD70, also known as TNFSF7 or the CD27 ligand, can combine with its receptor, CD27, and activate downstream responses, including the NF-*κ*B and Jun amino-terminal kinase pathways. At present, it is believed that CD70 is responsible for the immune evasion mechanism, which is similar to the effect of B7-CD28 families [22]. This may be another potential target for future immunotherapy. The preliminary antitumour capacity and good tolerability of ARGX-110, a type of anti-CD70 antibody, was demonstrated in a phase I study [23]. Yang et al. found that CD70 can drive tumour progression and cause immunosuppression in gliomas [24]. To be brief, high expression level of CD70 might indicate poor prognosis of gliomas.

Ectodysplasin A (EDA) has two isoforms, EDA-A1 and EDA-A2. The NF-*κ*B pathway is activated once EDA-A1 binds to EDAR, accompanied by the recruitment of TRAF1, TRAF3, and TRAF6. In patients with colorectal carcinoma and breast cancer, the EDA-A2-XEDAR interactions could induce the death of tumour cells, parallel with the decline in XEDAR expression [25–27]. Whether an analogous effect will emerge within diffuse gliomas is unknown and needs to be verified in further studies.

TNFRSF11B, also known as osteoprotegerin (OPG), is secreted mainly by osteoblast lineage cells. Researchers have found that OPG blocked the interaction between TNFRSF11A (RANK) and receptor activator of nuclear factor kappa-B ligand (RANKL) by acting as a decoy receptor [28]. It is acknowledged widely that the binding of RANK and RANKL activates the NF-*κ*B pathway, which plays an important role in the stem-like cell maintaining process, cellular proliferation and invasion, epithelial to mesenchymal transition process, and resistance to chemotherapy and radiotherapy in glioblastoma [29]. In addition, Kim et al. found that RANKL could reactivate the astrocytes, promote the cellular invasion, and might reshape the tumour microenvironment in gliomas [30]. From this perspective, TNFRSF11B may inactivate the RANK-RANKL pathway and further protect the patients with gliomas. Conversely, OPG was capable of binding the TNF-related apoptosis-inducing ligand (TRAIL) and thus hinder TRAIL from inducing apoptosis of tumour cells. This might attenuate the efficacy of Apo2L/TRAIL-based therapy in gliomas [31]. In our study, TNFRSF11B was found to be an independent risk factor in TCGA cohorts, and further investigation is needed.

TNFRSF14, more widely known as herpesvirus entry mediator (HVEM), has been deemed as a molecular switch showing both costimulatory and coinhibitory effects on T cells in various malignancies [32]. Hokuto et al. reported that overexpression of HVEM was frequently found in patients with hepatocellular carcinoma. High expression of HVEM was significantly linked to shortened OS and recurrence-free survival because of the paucity of tumour-infiltrating T cells and dysfunction of local immune responses [33]. In another study, a similar result was obtained from patients with glioblastoma, and the underlying mechanisms were not specifically illuminated [18].

TNFRSF25, also known as death receptor 3 (DR3), was a receptor primarily anchored on the surface of the T cell. It could mediate the process of apoptosis and differenciation [34–36]. Previous literature reported that TL1A (TNFSF15), the exclusive ligand of DR3, can increase the number of CD4+ effector T cells in inflammatory models and potentiate the activity of CD8+ T cells, Treg, and NKT cells [37–40]. Based on the murine model of plasmacytoma, Slebioda et al. demonstrated that overexpression of TL1A intrigued antitumour effect with the existence of CD8+ T cells. Moreover, they found the TNFRSF25 was capable of facilitating the proliferation and CTL-oriented differentiation of CD8+ T cells [40]. In our univariate and multivariate Cox regression analysis, TNFRSF25 was a protective factor, which preliminarily showed the probability that TNFRSF25 might incur the analogous antitumour effect in patients of diffuse gliomas. However, further investigation is needed to exemplify this hypothesis.

TNFRSF12A is also known as fibroblast growth factor-inducible 14 (Fn14). Hersh et al. identified that Fn14 was overexpressed in patient-derived xenograft cell lines isolated from recurrent glioblastoma and gliosarcomas in comparison with nonneoplastic brain tissues and primary glioblastomas. Of note, patients with high Fn14 mRNA expression showed shortened OS. Moreover, they found that specimens resected from patients who underwent temozolomide (TMZ) treatment had higher FN14 levels than those who did not. They further demonstrated that TMZ-resistant GBM cells had a better performance in terms of cellular migration than their TMZ-sensitive counterparts [41]. Tan et al. identified that overexpression of Fn14 is linked to poor glioma prognosis [42]. In our study, TNFRSF12A was identified as an independent risk factor in TCGA, CGGA, and GSE16011 cohorts by using multivariate Cox regression analysis. By incorporating the previous findings reported by Hersh et al., we hypothesised that targeting Fn14 would achieve a promising therapeutic effect in LGGs.

Our signature was robustly established in TCGA dataset and validated in three other public databases, namely, CGGA, GSE16011, and REMBRANDT. The Riskscore of each patient in these databases was calculated using the aforementioned LASSO model and confirmed as an independent risk factor in one training cohort and three validation cohorts with the aid of univariate and multivariate Cox regression analyses. Moreover, Kaplan–Meier survival analysis revealed that the OS of the high-risk group was significantly shorter than that of the low-risk group in these four databases. It is widely acknowledged that the IDH mutation status of gliomas could indicate the prognosis of a patient extensively according to the 2016 WHO guidelines [43]. In diffuse gliomas of all grades, patients with higher Riskscore were primarily distributed in the group without IDH mutation, which indicated that our signature had an analogous predictive accuracy of prognosis to that of IDH. Previous studies have indicated that the proneural transcriptome subtype has a more favourable prognosis than the mesenchymal subtype [44]. High-risk patients with LGGs were scattered across the mesenchymal subgroup, whereas a low Riskscore was more likely indicative of the proneural counterpart. Combined with the results of the ROC curves, we conclude that our signature precisely reflects the prognosis of patients with LGGs.

Regarding the results of the CIBERSORT, we found that the proportion of M0, M1, and M2 macrophages were significantly higher in the high-risk group, while the naïve B cells and naïve CD4+ T cells were significantly higher in the low-risk group in both TCGA and CGGA cohorts. It is considered that M2 macrophages are correlated with the suppressive tumour microenvironment while the M1 macrophages are opposite to that of the M2 macrophages [45–49]. Considering that the proportion of M0 and M1 macrophages is extremely scarce compared to that of M2 macrophages, we could reasonably infer that M2 macrophages play a major role in the tumour microenvironment. Naïve B cells could become plasma cells, germinal center (GC) B cells, and memory B cells. CD20(+) B cells are associated with enhanced tumour immunity and prolong the survival of patients with melanoma [50]. However, in another study, when a B cell stimulus *α*CD40 is implemented in a murine glioma model, suppressive CD11b + B cells are induced and downregulate the cytotoxic T cell responses [51]. In our study, the proportion of naïve B cells is higher in the low-risk group than that of the high-risk group in both databases. We hypothesise that a higher proportion of naïve B cells could probably lead to more functional B cell production. Another explanation is that more naïve B cells differentiate towards immunosuppressive phenotype in the high-risk group. However, the specific function of naïve B cells in gliomas is unclear. Su et al. found that the circulating naïve CD4(+) T cells can differentiate to Treg in breast cancer [52]. Likewise, the correlation between naïve CD4(+) T cells and gliomas is unclear and further investigation is needed. Concurrently, we found that the Riskscore had positive linear correlations with the expression of immune checkpoint molecules. These results indicate that our signature might somehow be linked with the exhausting phenotype of various cells. In addition, we found that the mutated genes and pathways differed in the high-risk and low-risk groups, especially IDH1, FUBP1, CIC, and EGFR. The biological pathways involved were the RTK-RAS, PI3K, and TP53 pathways. This showed that our signature might be correlated with specific genes and pathways that regulate the growth and proliferation of tumour cells.

Hersh et al. reported that overexpression of the IDH1 R132H protein (a common form of mutant IDH1) reduced the expression of Fn14 (TNFRSF12A) in gliomas [53]. However, the underlying mechanism has not been explained. In our analysis, patients with higher Riskscore had worse life expectancy and were less likely to harbour the IDH1 mutation. The homolog of the Drosophila capicua (CIC) and its far upstream binding protein 1 (FUBP1) are located on the 19q and 1p chromosomal arms, respectively. CIC and FUBP1 mutations occur probably because of the unbalanced translocation and often emerge after IDH mutation and 1p/19q codeletion in gliomas [54]. It is widely acknowledged that IDH mutated status and 1p/19q codeletion status are vital prognostic factors of diffuse gliomas; therefore, the appearance of these two mutated genes along with IDH mutation and 1p/19q codeletion might similarly infer a good prognosis of gliomas. This is in line with our result that the occurrence of CIC and FUBP1 mutation in the low-risk group is much higher than that in the high-risk group. CIC is located on the upstream of RTK-RAS pathway and might downregulate this critical oncogenesis pathway [55]. No reports have elucidated the function of FUBP1 mutation in gliomas. These genetic changes have been discovered with the recent popularity of genome sequencing; therefore, the specific function of the two mutated genes in gliomas remains unclear and more investigations are needed.

Previous studies have identified that augmentation and mutations of EGFR play a critical role in tumorigenesis, and patients suffering from lung cancer harbouring this mutation or amplification benefited most from EGFR tyrosine kinase inhibitors (TKIs) [56, 57]. In general, 40–50% of patients with glioblastomas also show overexpression and augmentation of EGFR. Nevertheless, the therapeutic effect of EGFR TKIs has not yet been reported [58–60], and Guo et al. found that TNF and EGFR inhibitors had a synergistic effect in TMZ-resistant glioblastoma. The obstruction of EGFR signalling stimulated the secretion of TNF and subsequently activated the surviving pathways. Therefore, blocking the TNF-related surviving pathways could sensitise glioblastomas to TMZ [5, 6]. In our study, patients with higher Riskscore tended to harbour EGFR mutations. We hypothesised that TNF-related survival pathways might be an alternative approach for gliomas to escape the lethality of various types of treatments, but more evidence is needed.

It is widely acknowledged that activation of receptor-linked tyrosine kinases (RTKs) and the downstream RAS pathway can lead to uncontrolled proliferation of all malignancies [61]. TP53 is one of the most critical tumour-suppressive genes in humans. If TP53 expression is compromised, cell division will go haywire, and nearly half of all human malignancies harbour TP53 alterations [62]. PI3K/AKT/mTOR pathway activation has been observed in various cancers because of its capacity to inhibit apoptosis and promote cellular proliferation.

The aforementioned findings could be further evidenced by GSEA and GO analysis. In TCGA and CGGA cohorts, KRAS signalling, TP53 pathway, and PI3K/AKT/mTOR signalling were enriched in the high-risk group. In addition, epithelial to mesenchymal transition and positive regulation of I-*κ*B kinase/NF-*κ*B signalling were identified using GSEA and GO analysis. Numerous documents have recorded that epithelial to mesenchymal transition (EMT) is linked to the invasive features of gliomas which enable it to penetrate the adjacent stroma cells. In addition, EMT contributes to the immunosuppressive microenvironment conducive to the progression and metastasis of gliomas [63, 64]. Abnormal activation of the NF-*κ*B signalling pathway usually promotes the malignant cell proliferation and invasion, suppresses the tumour immune response, and leads to chemotherapy resistance [29, 65, 66]. In summary, we hypothesise that the enrichment of NF-*κ*B signalling and EMT pathways in TNF-based high-risk group leads to cell proliferation and invasion and the formation of immunosuppressive microenvironment, which shortens the patients OS, causing recurrence and therapy resistance.

In this study, we first established a robust prognostic model in LGGs based on the TNF superfamily and preliminarily confirm the predicting value of these immune-related cytokines. Meanwhile, we have found that high-risk subjects of our prognostic model are correlated with higher expression levels of immune checkpoint genes, higher proportion of M2 macrophages, and the hyperactive RTK-RAS pathway and innovatively propose that TNF is involved with the formation of immunosuppressive microenvironment, and the malignant proliferation process of LGGs. In the future, it may provide new drug targets for inhibiting the tumour growth, attenuating the immune checkpoint therapy resistance, and reversing the immunosuppressive microenvironment. However, this is a retrospective study, and more evidence is needed to further support these analyses.

## Figures and Tables

**Table 1:** Demographic statistics and clinical features of grade II/III patient cohort.

Character	TCGA (*n* = 449)	CGGA (*n* = 443)	GSE16011 (*n* = 108)	REMBRANDT (*n* = 143)
Age				
<45	262 [58.4%]	297 [67.0%]	60 [55.6%]	—
≥45	187 [41.6%]	145 [32.7%]	48 [44.4%]	—
NA	0 [0%]	1 [0.3%]	0 [0%]	143 [100%]
Sex				
Male	251 [55.9%]	251 [56.7%]	—	65 [45.5%]
Female	198 [44.1%]	192 [43.3%]	—	41 [28.6%]
NA	0 [0%]	0 [0%]	108 [100%]	37 [25.9%]
WHO classification				
WHO II	213 [47.4%]	188 [42.4%]	23 [21.3%]	72 [50.3%]
WHO III	236 [52.6%]	255 [57.6%]	85 [78.7%]	71 [49.7%]
IDH mutation status				
Wildtype	84 [18.7%]	96 [21.7%]	40 [37.0%]	—
Mutant	363 [80.8%]	306 [69.1%]	47 [43.5%]	—
NA	2 [0.5%]	41 [9.2%]	21 [19.5%]	143 [100%]
1p/19q codeletion status				
Noncodeletion	300 [66.8%]	273 [61.6%]	40 [37.0%]	—
Codeletion	149 [33.2%]	131 [29.6%]	38 [35.2%]	—
NA	0 [0%]	39 [8.8%]	30 [27.8%]	143 [100%]
Overall survival (months)				
Median	87.394	83.700	41.640	42.60
Range	0.033-211.027	1.7-167.6	0.24-248.16	0.2-251.733
Survival rates (years)				
1	0.93	0.88	0.82	0.77
2	0.86	0.76	0.62	0.60
5	0.67	0.56	0.40	0.39

“-” symbol indicates that the statistics were unavailable in the corresponding database. NA: not available.

## Data Availability

The data used in this study were downloaded from the official website of TCGA (https://cancergenome.nih.gov/), CGGA (http://www.cgga.org.cn/), GSE16011 (https://www.ncbi.nlm.nih.gov/), and REMBRANDT (http://www.betastasis.com/glioma/rembrandt/).

## Abbreviation

CGGA: Chinese Glioma Genome Atlas
CTLA-4: Cytotoxic T-lymphocyte-associated protein 4
CTLs: Cytotoxic T-lymphocytes
EGFR: Epidermal growth factor receptor
GO: Gene Ontology
HR: Hazard ratio
IDH: Isocitrate dehydrogenase
LAG-3: Lymphocyte-activation gene 3
LASSO: Least absolute shrinkage and selection operator
LGG: Lower-grade gliomas
MGMT: Methylguanine methyltransferase
OS: Overall survival
PD-1: Programmed death receptor 1
PD-L1: Programmed death receptor 1 ligand
TCGA: The Cancer Genome Atlas
TGF-*β*: Transforming growth factor-beta
TIM-3: T cell immunoglobulin and mucin domain 3
TMB: Tumour mutation burden
TNF: Tumour necrosis factor
TNFSF/TNFRSF: TNF and TNF receptor (TNFR) superfamilies
95% CI: 95% confidence interval
PFS: Progression-free survival
RFS: Recurrence-free survival
RTK: Receptor-linked tyrosine kinase
TAA: Tumour-associated antigen
TKI: Tyrosine kinase inhibitor
TMZ: Temozolomide
WHO: World Health Organization.

## Additional Points

*Highlights*. (i) A six-gene signature based on TNFSF/TNFRSF accurately predicts the prognosis of lower-grade gliomas (LGG). (ii) This six-gene signature correlated significantly with the expression of immune checkpoint genes and the infiltrating M2 macrophages in tumour specimens. (iii) TNFSF/TNFRSF might influence the LGG prognosis through specific types of tumour mutation, which could be targeted effectively.

## Supplementary Materials

Supplementary 1 Supplementary Table 1: result of Lasso Cox analysis. Supplementary Table 2: Cox regression analysis of clinical characteristics and six gene-based Riskscore in the TCGA cohort. Supplementary Table 3: Cox regression analysis of clinical characteristics and six gene-based Riskscore in the CGGA cohort. Supplementary Table 4: Cox regression analysis of clinical, gene expression characteristics, and six gene-based Riskscore in the GSE16011 cohort. Supplementary Table 5: correlation between Riskscore and gene expression levels of immune checkpoint-related genes in the TCGA. Supplementary Table 6: correlation between Riskscore and gene expression levels of immune checkpoint-related genes in the CGGA.

Supplementary 2 Supplementary Figure 1: (a) nomogram (up) and its calibration plots (down) for predicting LGG prognosis in the TCGA cohort. (b) Nomogram (up) and its calibration plots (down) for predicting LGG prognosis in the CGGA cohort. (c) Nomogram (up) and its calibration plots (down) for predicting LGG prognosis in the GSE16011 cohort.

Supplementary 3 Supplementary Figure 2: Gene Ontology analysis demonstrating the biological processes related to Riskscore in the GSE16011 (a) and REMBRANDT (b) dataset.

Supplementary 4 Supplementary Figure 3: GSEA analysis of data from the TCGA (a), CGGA (b), GSE16011 (c), and REMBRADANT (d) database showing the pathway enriched in the high-risk group. The nominal *p* value is less than 0.05, and the FDR *q* value is less than 0.25 in all figures. In each GSEA graph, the green curves in the upper panels represent the enrichment score (ES) of each gene in the ranking list. The black bars in the middle panels mean the position of each gene from a settled gene set (e.g., HALLMARK_ANGIOGENESIS) in the ranking gene list. The red gradients means the corresponding genes overexpress in high risk group while the blue gradients overexpress in the low risk group. The grey shading in the lower panels refers to signal-to-noise ratio of each gene inputted into the GSEA software, and the genes aforementioned are ranked from highest to lowest.
