# Supplementary material for: A Novel TNFSF-Based Signature Predicts the Prognosis and Immunosuppressive Status of Lower-Grade Glioma
Source: Biomed Res Int. 2022 May 9;2022:3194996. doi: 10.1155/2022/3194996 (PMC9112166; doi:10.1155/2022/3194996)
Supplement: Supplementary 1 — Supplementary Table 1: result of Lasso Cox analysis. Supplementary Table 2: Cox regression analysis of clinical characteristics and six gene-based Riskscore in the TCGA cohort. Supplementary Table 3: Cox regression analysis of clinical characteristics and six gene-based Riskscore in the CGGA cohort. Supplementary Table 4: Cox regression analysis of clinical, gene expression characteristics, and six gene-based Riskscore in the GSE16011 cohort. Supplementary Table 5: correlation between Riskscore and gene expression levels of immune checkpoint-related genes in the TCGA. Supplementary Table 6: correlation between Riskscore and gene expression levels of immune checkpoint-related genes in the CGGA. [file 3194996.f1.pdf]

Supplementary table 1 Result of Lasso Cox analysis

Gene	Coefficient
CD70	0.128514092
EDA	-0.061112803
TNFRSF11B	0.463397817
TNFRSF12A	0.27958787
TNFRSF14	0.015476169
TNFRSF25	-0.029856055

Supplementary table 2 Cox regression analysis of clinical characteristics and six gene-based Riskscore in the TCGA cohort

	Univariate analysis			Multivariate analysis		
	HR	95 % CI	P value	HR	95 % CI	P value
Age	1.066	1.047-1.085	<0.0001	1.064	1.044-1.085	<0.0001
IDH status	0.157	0.098-0.251	<0.0001	0.287	0.139-0.595	0.001
1p/19q co-deletion	0.422	0.240-0.741	0.003	0.460	0.246-0.861	0.015
MGMT promoter status	0.430	0.263-0.704	0.001			
Grade	3.271	1.996-5.361	<0.0001	2.182	1.293-3.684	0.003
TNFRSF11B	2.477	1.968-3.119	<0.0001	1.755	1.189-2.590	0.005
TNFRSF12A	2.838	2.136-3.770	<0.0001	2.129	1.421-3.191	<0.0001
Riskscore	4.496	3.206-6.304	<0.0001	3.483	2.011-6.034	<0.0001

IDH status: 0-wildtype, 1-mutated. 1p/19q co-deletion: 0-non co-deletion, 1-co-deletion. MGMT promoter status: 0-unmethylated, 1-methylated. Grade: 2-WHO II, 3-WHO III. The absence of values in the table means the corresponding variable is not an independent risk factor ($p > 0.05$) in multivariate analysis.

Supplementary table 3 Cox regression analysis of clinical characteristics and six gene-based Riskscore in the CGGA cohort

	Univariate analysis			Multivariate analysis		
	HR	95 % CI	P value	HR	95 % CI	P value
IDH status	0.471	0.346-0.642	<0.0001	0.581	0.411-0.821	0.002
1p/19q co-deletion	0.357	0.247-0.518	<0.0001	0.430	0.287-0.644	<0.0001
Chemotherapy	1.084	0.793-1.482	0.612			
Radiotherapy	1.299	0.912-1.850	0.147			
Grade	2.634	1.908-3.636	<0.0001	3.227	2.251-4.625	<0.0001
TNFRSF11B	1.242	1.112-1.387	<0.0001	1.122	0.987-1.275	0.078
TNFRSF12A	2.045	1.693-2.471	<0.0001	1.488	1.192-1.856	<0.0001
Riskscore	1.575	1.336-1.857	<0.0001	1.334	1.100-1.617	0.003

IDH status: 0-wildtype, 1:-mutated. 1p/19q co-deletion: 0-non co-deletion, 1-co-deletion. Chemotherapy: 0-untreated, 1-treated. Radiotherapy: 0-untreated, 1-treated. Grade: 2-WHO II ,3-WHO III. The absence of values in the table means the corresponding variable is not an independent risk factor ($p > 0.05$) in multivariate analysis.

Supplementary table 4 Cox regression analysis of clinical , gene expression characteristics, and six gene-based Riskscore in the GSE16011 cohort

	Univariate analysis			Multivariate analysis		
	HR	95 % CI	P value	HR	95 % CI	P value
Age	1.035	1.017-1.053	<0.0001	1.032	1.004-1.061	0.025
EGFR status	5.333	2.497-11.386	<0.0001			
1p/19q co-deletion	0.485	0.293-0.804	0.005	0.387	0.183-0.821	0.013
IDH status	0.826	0.514-1.328	0.430			
Chemotherapy	1.026	0.568-1.854	0.932			
KPS	0.980	0.971-0.989	<0.0001			
Grade	1.190	0.679-2.086	0.542			
TNFRSF11B	1.430	1.186-1.723	<0.0001			
TNFRSF12A	2.172	1.606-2.937	<0.0001	2.400	1.215-4.742	0.012
TNFRSF14	1.505	1.187-1.908	0.001	1.742	1.031-2.942	0.038
Riskscore	2.516	1.777-3.562	<0.0001	2.192	1.098-4.376	0.026

IDH status:0: wildtype, 1: mutation. 1p/19q: co-deletion: 0: non co-deletion: 1: co-deletion. MGMT promoter status: 0: unmethylated,1: methylated. Grade: 2: WHO II ,3: WHO III. The absence of values in the table means the corresponding variable is not an independent risk factor (p > 0.05) in multivariate analysis.

Supplementary table 5 Correlation between Riskscore and gene expression levels of immune checkpoint-related genes in the TCGA

	Pearson correlation coefficient	P value
PD-1	0.553	< 0.0001
PD-L1	0.525	< 0.0001
CTLA-4	0.400	< 0.0001
TIM-3	0.463	< 0.0001
LAG-3	0.149	0.002
TGF-β	0.438	< 0.0001

Supplementary table 6 Correlation between Riskscore and gene expression levels of immune checkpoint-related genes in the CGGA

	Pearson correlation coefficient	P value
PD-1	0.231	< 0.0001
PD-L1	0.228	< 0.0001
CTLA-4	0.146	0.002
TIM-3	0.344	< 0.0001
LAG-3	-0.031	0.522
TGF-β	0.335	< 0.0001
