# Supplementary material for: A Novel TNFSF-Based Signature Predicts the Prognosis and Immunosuppressive Status of Lower-Grade Glioma
Source: Biomed Res Int. 2022 May 9;2022:3194996. doi: 10.1155/2022/3194996 (PMC9112166; doi:10.1155/2022/3194996)
Supplement: Supplementary 2 — Supplementary Figure 1: (a) nomogram (up) and its calibration plots (down) for predicting LGG prognosis in the TCGA cohort. (b) Nomogram (up) and its calibration plots (down) for predicting LGG prognosis in the CGGA cohort. (c) Nomogram (up) and its calibration plots (down) for predicting LGG prognosis in the GSE16011 cohort. [file 3194996.f2.pdf]

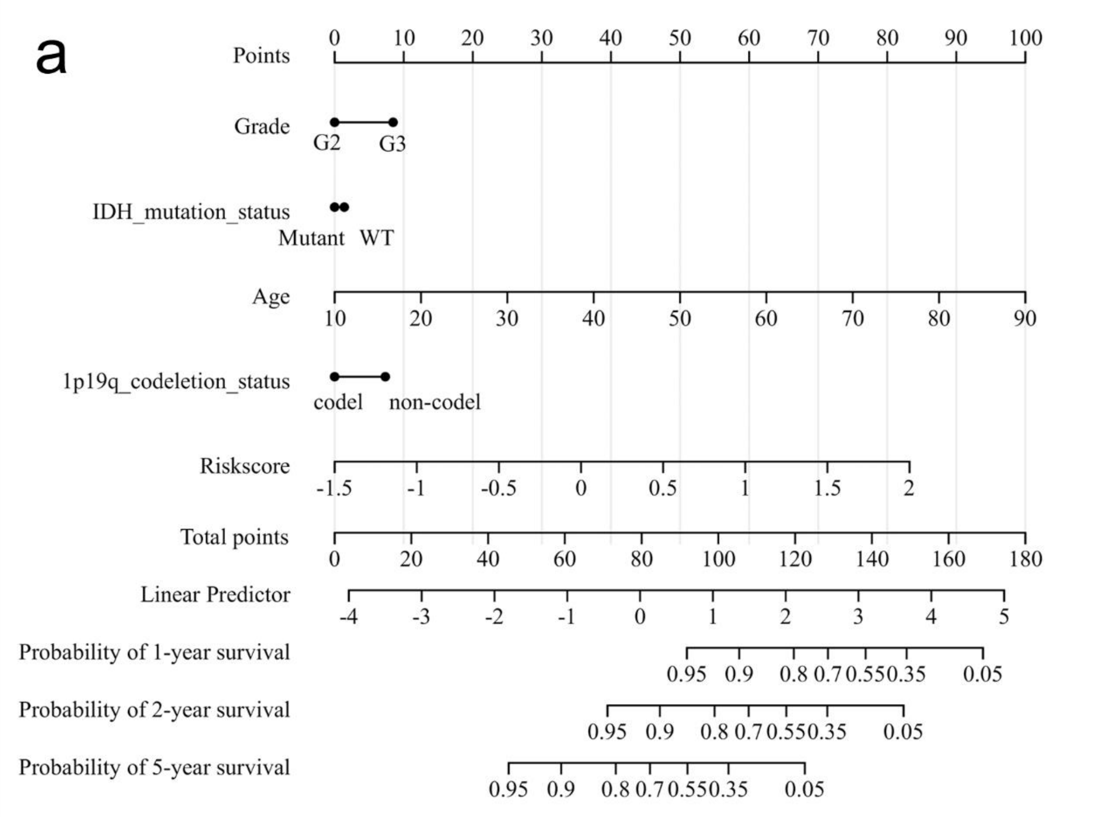

a
Points
0
10
20
30
40
50
60
70
80
90
100
Grade
G2
G3
IDH_mutation_status
Mutant
WT
Age
10
20
30
40
50
60
70
80
90
1p19q_codeletion_status
codel
non-codel
Riskscore
-1.5
-1
-0.5
0
0.5
1
1.5
2
Total points
0
20
40
60
80
100
120
140
160
180
Linear Predictor
-4
-3
-2
-1
0
1
2
3
4
5
Probability of 1-year survival
0.95 0.9 0.8 0.7 0.55 0.35 0.05
Probability of 2-year survival
0.95 0.9 0.8 0.7 0.55 0.35 0.05
Probability of 5-year survival
0.95 0.9 0.8 0.7 0.55 0.35 0.05

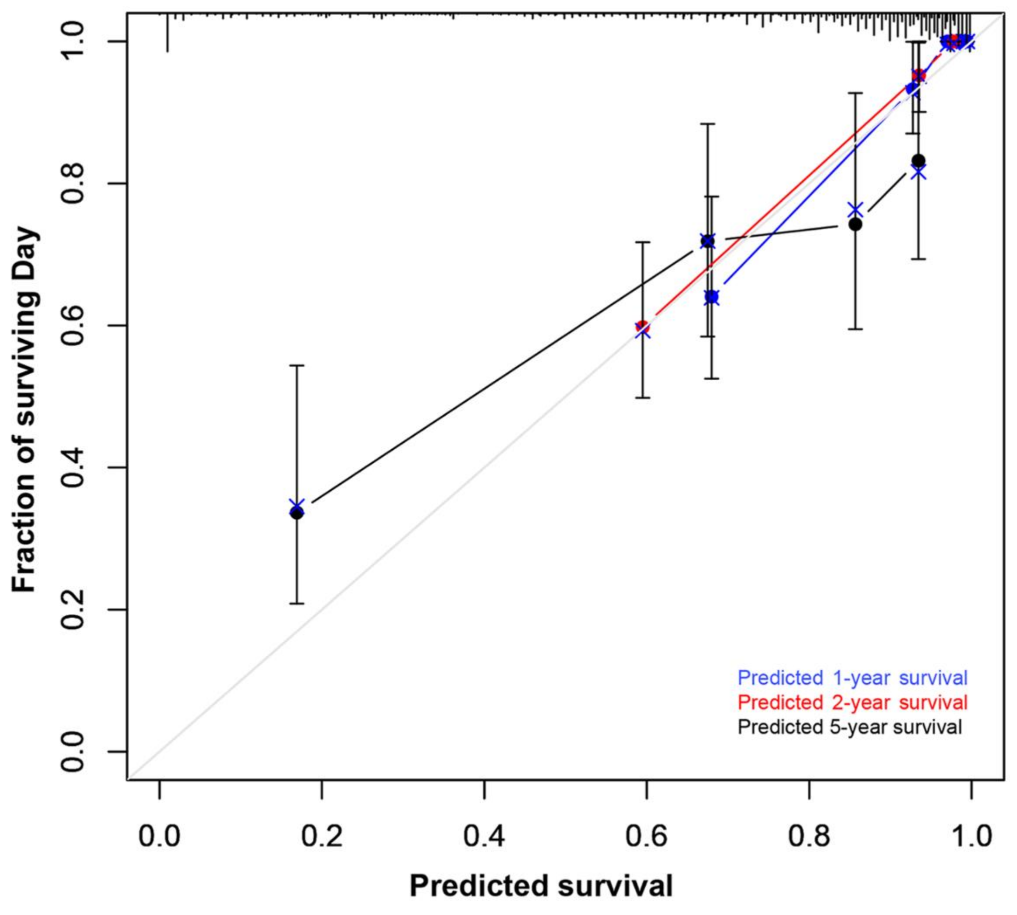

Fraction of surviving Day
0.0
0.2
0.4
0.6
0.8
1.0
Predicted survival
0.0
0.2
0.4
0.6
0.8
1.0
Predicted 1-year survival
Predicted 2-year survival
Predicted 5-year survival

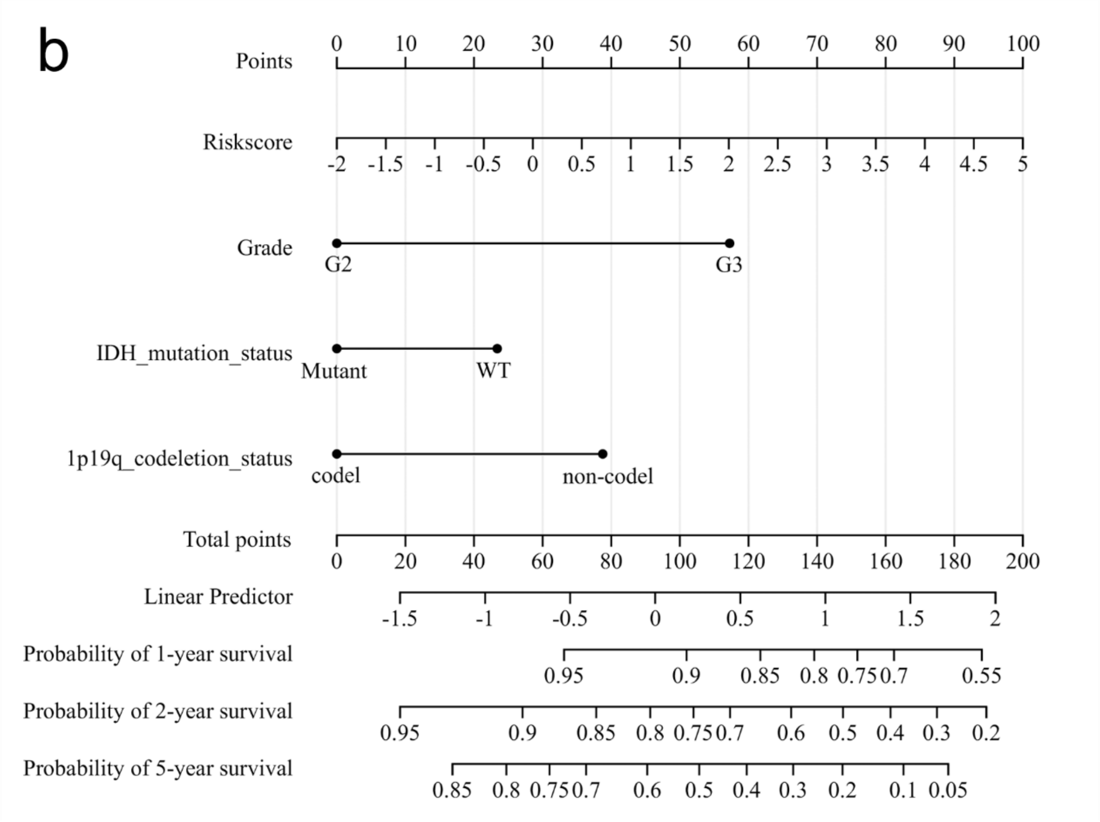
b
Points
0 10 20 30 40 50 60 70 80 90 100
Riskscore
-2 -1.5 -1 -0.5 0 0.5 1 1.5 2 2.5 3 3.5 4 4.5 5
Grade
G2
G3
IDH_mutation_status
Mutant
WT
1p19q_codeletion_status
codel
non-codel
Total points
0 20 40 60 80 100 120 140 160 180 200
Linear Predictor
-1.5 -1 -0.5 0 0.5 1 1.5 2
Probability of 1-year survival
0.95 0.9 0.85 0.8 0.75 0.7 0.55
Probability of 2-year survival
0.95 0.9 0.85 0.8 0.75 0.7 0.6 0.5 0.4 0.3 0.2
Probability of 5-year survival
0.85 0.8 0.75 0.7 0.6 0.5 0.4 0.3 0.2 0.1 0.05

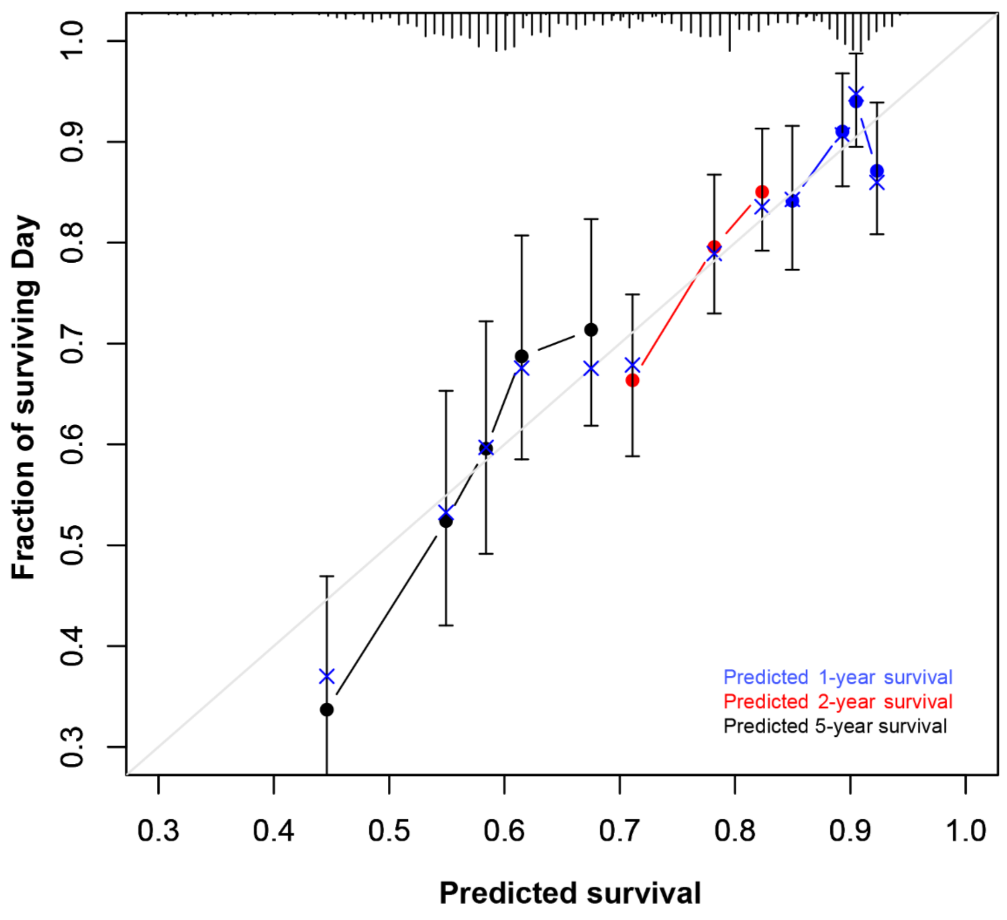
Fraction of surviving Day
0.3 0.4 0.5 0.6 0.7 0.8 0.9 1.0
Predicted survival
0.3 0.4 0.5 0.6 0.7 0.8 0.9 1.0
Predicted 1-year survival
Predicted 2-year survival
Predicted 5-year survival

C

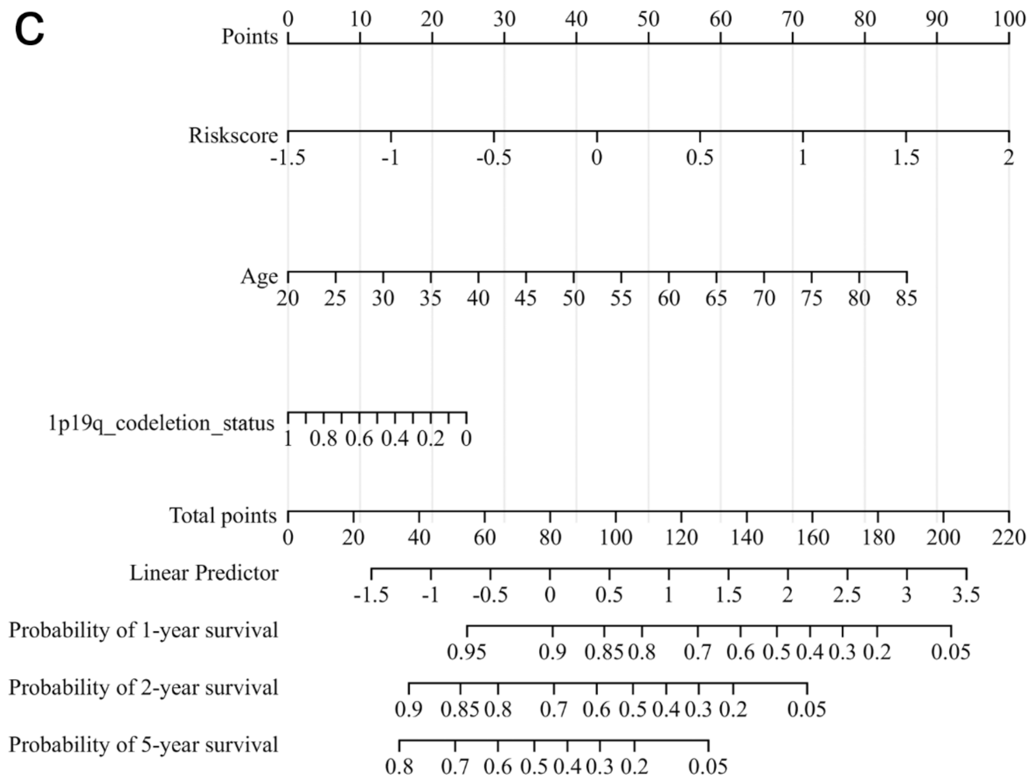
Points
0 10 20 30 40 50 60 70 80 90 100
Riskscore
-1.5 -1 -0.5 0 0.5 1 1.5 2
Age
20 25 30 35 40 45 50 55 60 65 70 75 80 85
1p19q_codeletion_status
1 0.8 0.6 0.4 0.2 0
Total points
0 20 40 60 80 100 120 140 160 180 200 220
Linear Predictor
-1.5 -1 -0.5 0 0.5 1 1.5 2 2.5 3 3.5
Probability of 1-year survival
0.95 0.9 0.85 0.8 0.7 0.6 0.5 0.4 0.3 0.2 0.05
Probability of 2-year survival
0.9 0.85 0.8 0.7 0.6 0.5 0.4 0.3 0.2 0.05
Probability of 5-year survival
0.8 0.7 0.6 0.5 0.4 0.3 0.2 0.05

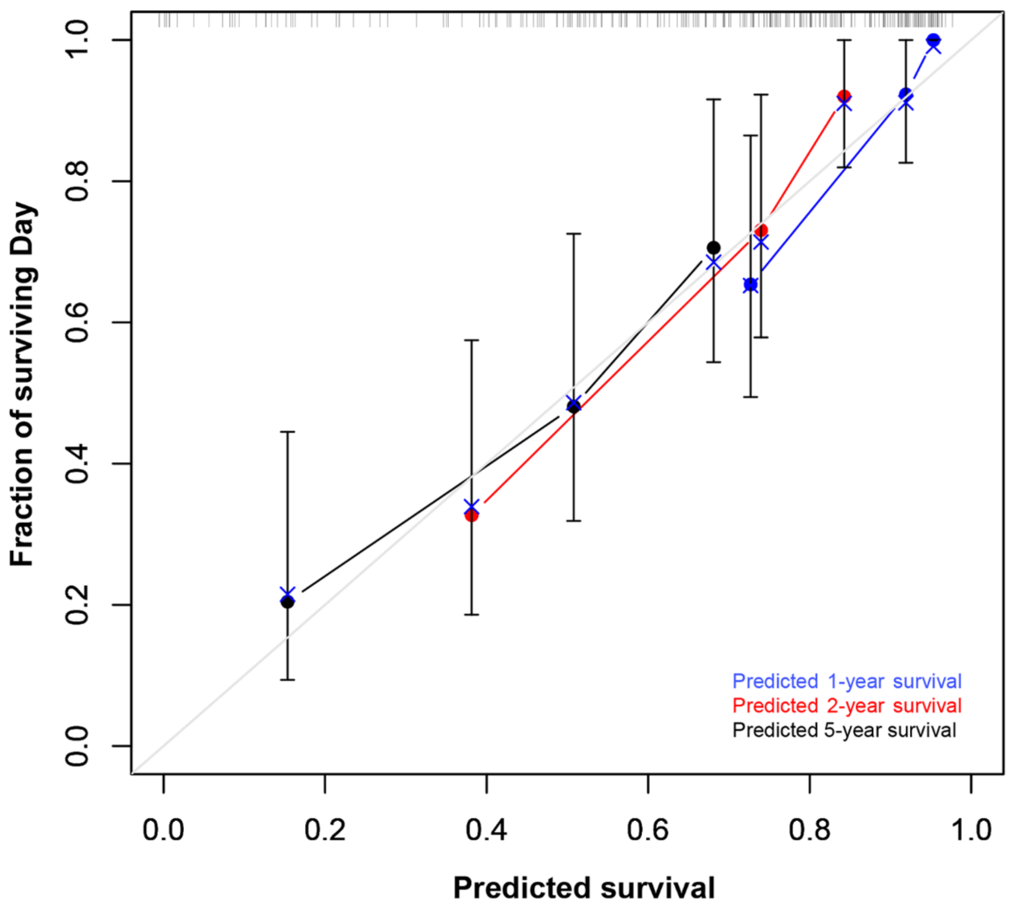
Fraction of surviving Day
0.0
0.2
0.4
0.6
0.8
1.0
Predicted survival
0.0 0.2 0.4 0.6 0.8 1.0
Predicted 1-year survival
Predicted 2-year survival
Predicted 5-year survival

Supplementary Fig 1. **a**, Nomogram (up) and its calibration plots (down) for predicting LGG prognosis in the TCGA cohort. **b**, Nomogram (up) and its calibration plots (down) for predicting LGG prognosis in the CGGA cohort. **c**, Nomogram (up) and its calibration plots (down) for predicting LGG prognosis in the GSE16011 cohort.
