# Supplementary material for: A Novel TNFSF-Based Signature Predicts the Prognosis and Immunosuppressive Status of Lower-Grade Glioma
Source: Biomed Res Int. 2022 May 9;2022:3194996. doi: 10.1155/2022/3194996 (PMC9112166; doi:10.1155/2022/3194996)
Supplement: Supplementary 3 — Supplementary Figure 2: Gene Ontology analysis demonstrating the biological processes related to Riskscore in the GSE16011 (a) and REMBRANDT (b) dataset. [file 3194996.f3.pdf]

a

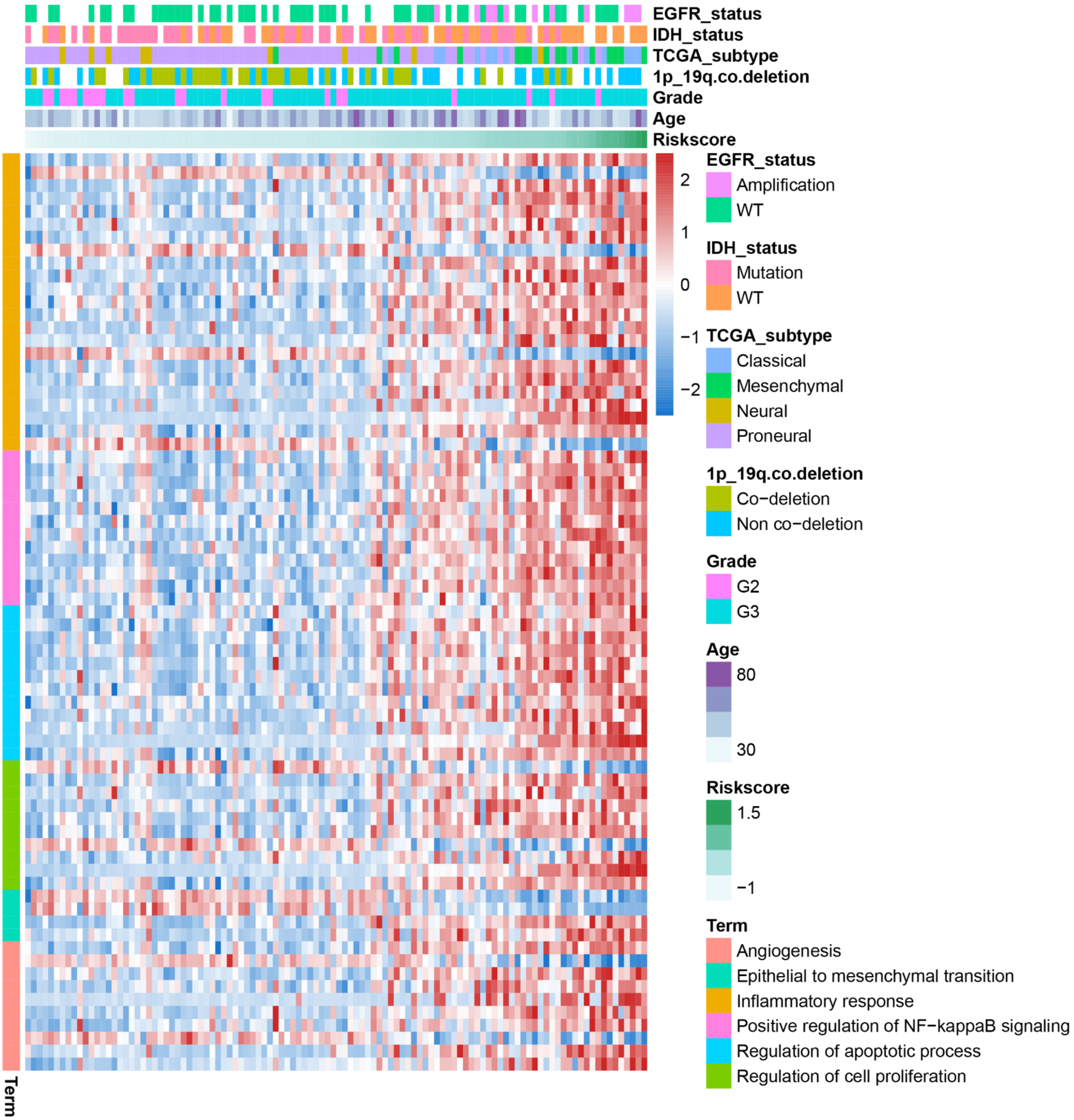

EGFR_status
IDH_status
TCGA_subtype
1p_19q.co.deletion
Grade
Age
Riskscore
2
1
0
−1
−2
EGFR_status
Amplification
WT
IDH_status
Mutation
WT
TCGA_subtype
Classical
Mesenchymal
Neural
Proneural
1p_19q.co.deletion
Co−deletion
Non co−deletion
Grade
G2
G3
Age
80
30
Riskscore
1.5
−1
Term
Angiogenesis
Epithelial to mesenchymal transition
Inflammatory response
Positive regulation of NF−kappaB signaling
Regulation of apoptotic process
Regulation of cell proliferation
Term

b

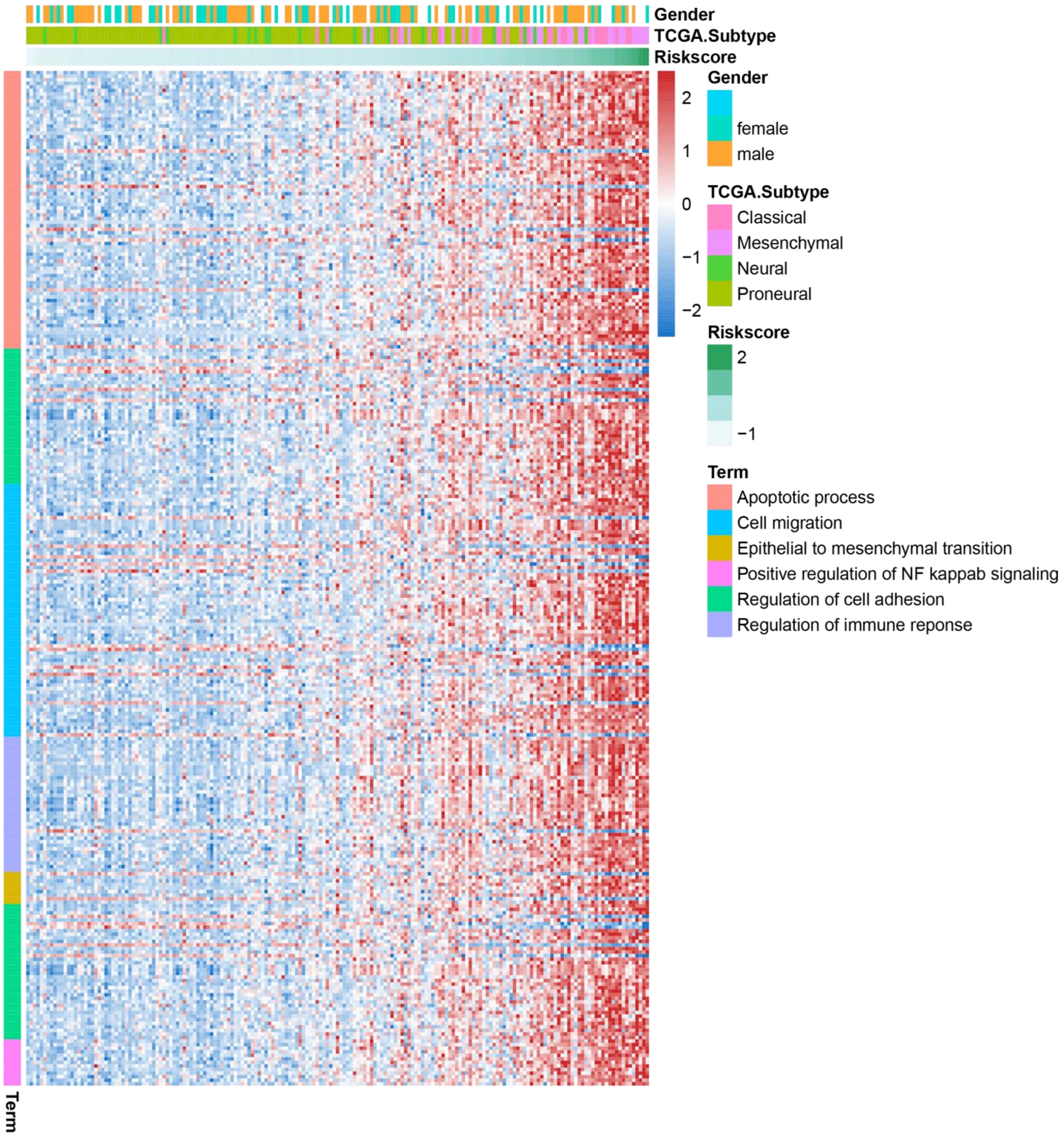
Gender
TCGA.Subtype
Riskscore
2
1
0
−1
−2
Gender
female
male
TCGA.Subtype
Classical
Mesenchymal
Neural
Proneural
Riskscore
2
−1
Term
Apoptotic process
Cell migration
Epithelial to mesenchymal transition
Positive regulation of NF kappab signaling
Regulation of cell adhesion
Regulation of immune reponse
Term

Supplementary Fig 2 Gene ontology analysis demonstrating the biological processes related to Riskscore in the GSE16011 (**a**) and REMBRANDT (**b**) dataset.
