# Supplementary material for: A Novel TNFSF-Based Signature Predicts the Prognosis and Immunosuppressive Status of Lower-Grade Glioma
Source: Biomed Res Int. 2022 May 9;2022:3194996. doi: 10.1155/2022/3194996 (PMC9112166; doi:10.1155/2022/3194996)
Supplement: Supplementary 4 — Supplementary Figure 3: GSEA analysis of data from the TCGA (a), CGGA (b), GSE16011 (c), and REMBRADANT (d) database showing the pathway enriched in the high-risk group. The nominal p value is less than 0.05, and the FDR q value is less than 0.25 in all figures. In each GSEA graph, the green curves in the upper panels represent the enrichment score (ES) of each gene in the ranking list. The black bars in the middle panels mean the position of each gene from a settled gene set (e.g., HALLMARK_ANGIOGENESIS) in the ranking gene list. The red gradients means the corresponding genes overexpress in high risk group while the blue gradients overexpress in the low risk group. The grey shading in the lower panels refers to signal-to-noise ratio of each gene inputted into the GSEA software, and the genes aforementioned are ranked from highest to lowest. [file 3194996.f4.pdf]

a

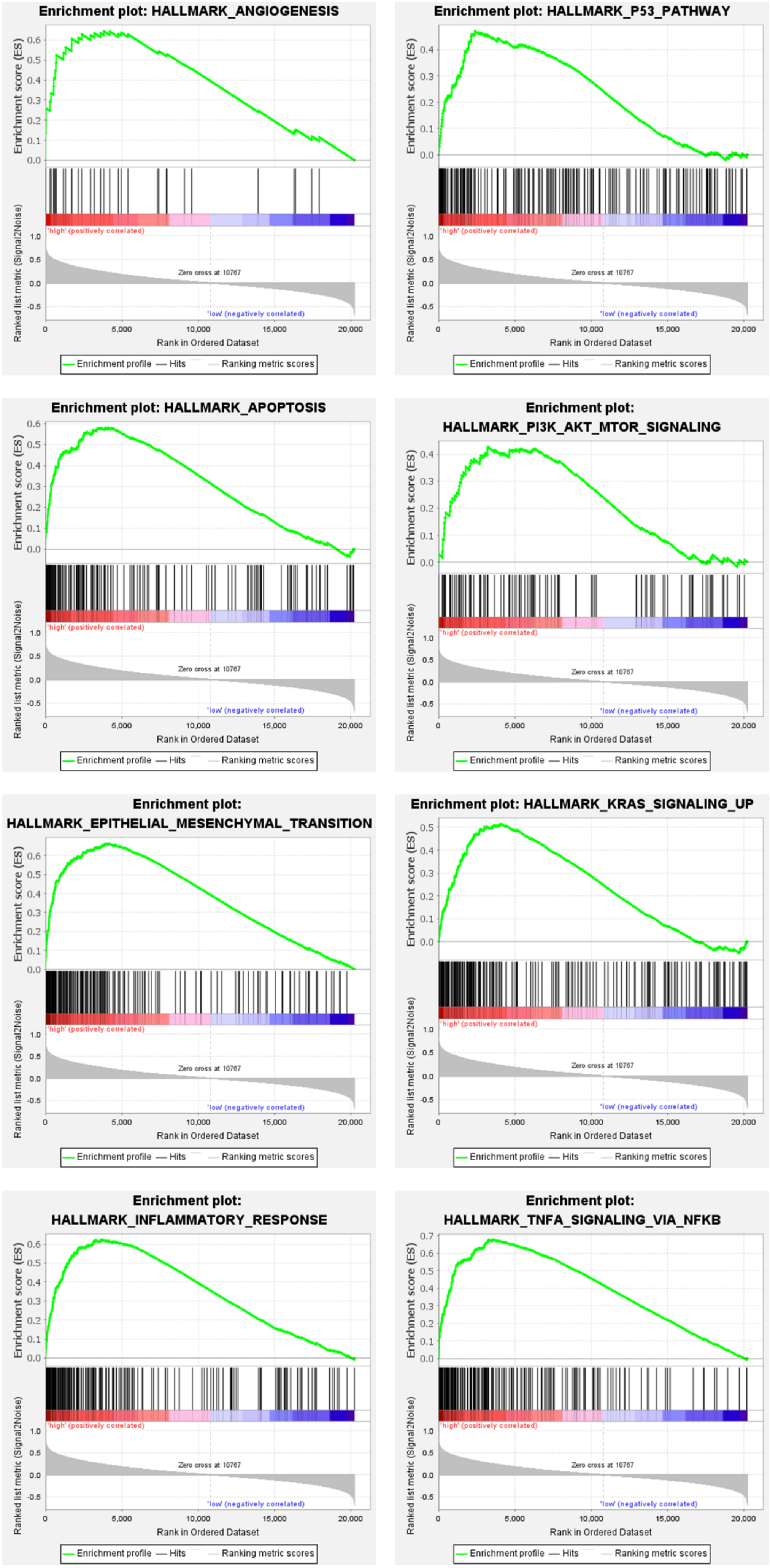

Enrichment plot: HALLMARK_ANGIOGENESIS
Enrichment plot: HALLMARK_P53_PATHWAY
Enrichment plot: HALLMARK_APOPTOSIS
Enrichment plot: HALLMARK_PI3K_AKT_MTOR_SIGNALING
Enrichment plot: HALLMARK_EPITHELIAL_MESENCHYMAL_TRANSITION
Enrichment plot: HALLMARK_KRAS_SIGNALING_UP
Enrichment plot: HALLMARK_INFLAMMATORY_RESPONSE
Enrichment plot: HALLMARK_TNFA_SIGNALING_VIA_NFKB
Enrichment score (ES)
Ranked list metric (Signal2Noise)
'high' (positively correlated)
Zero cross at 10767
'low' (negatively correlated)
Rank in Ordered Dataset
Enrichment profile — Hits — Ranking metric scores

b

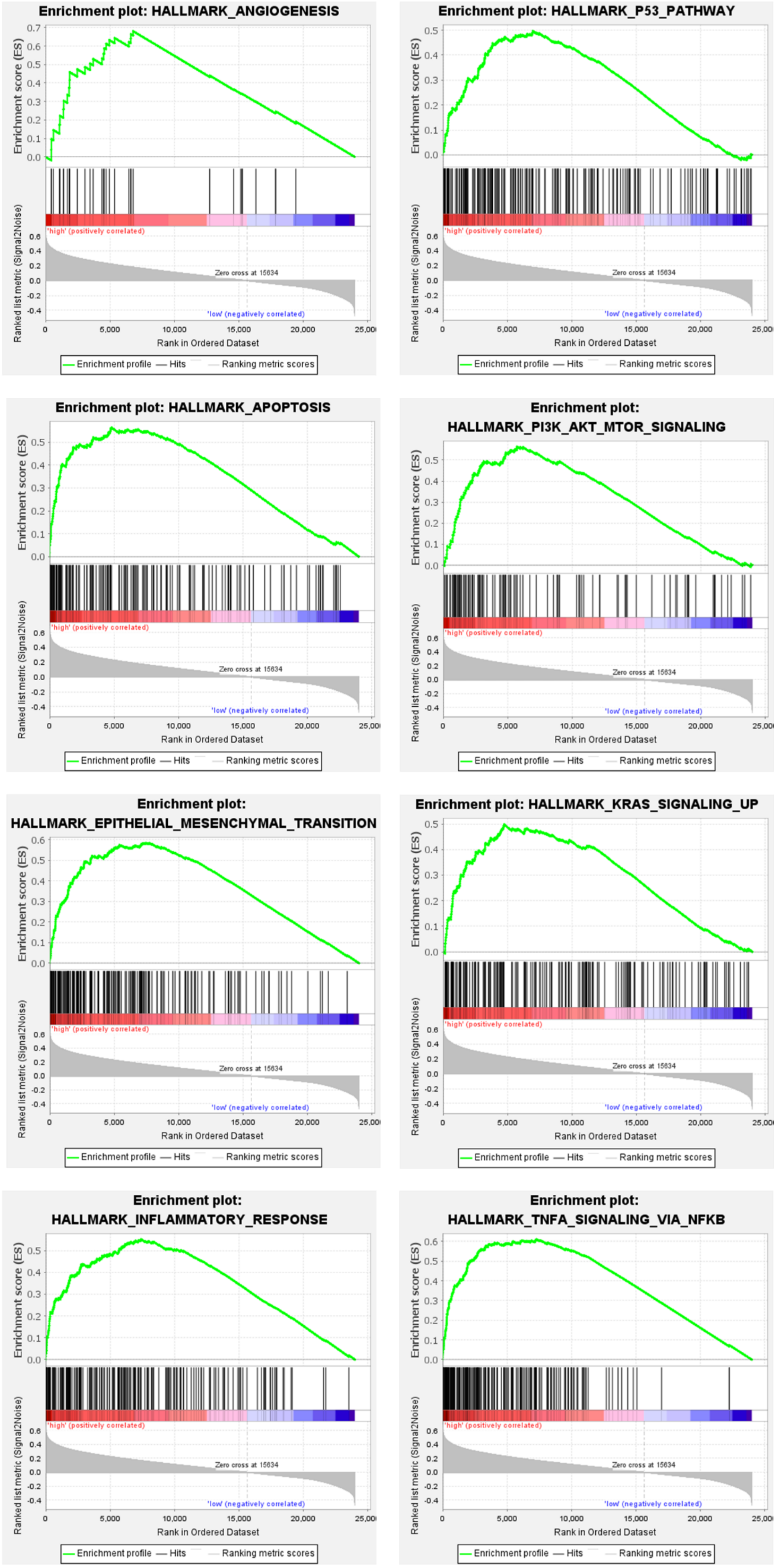
Enrichment plot: HALLMARK_ANGIOGENESIS
Enrichment plot: HALLMARK_P53_PATHWAY
Enrichment plot: HALLMARK_APOPTOSIS
Enrichment plot: HALLMARK_PI3K_AKT_MTOR_SIGNALING
Enrichment plot: HALLMARK_EPITHELIAL_MESENCHYMAL_TRANSITION
Enrichment plot: HALLMARK_KRAS_SIGNALING_UP
Enrichment plot: HALLMARK_INFLAMMATORY_RESPONSE
Enrichment plot: HALLMARK_TNFA_SIGNALING_VIA_NFKB
Enrichment score (ES)
Ranked list metric (Signal2Noise)
Rank in Ordered Dataset
Enrichment profile
Hits
Ranking metric scores

C

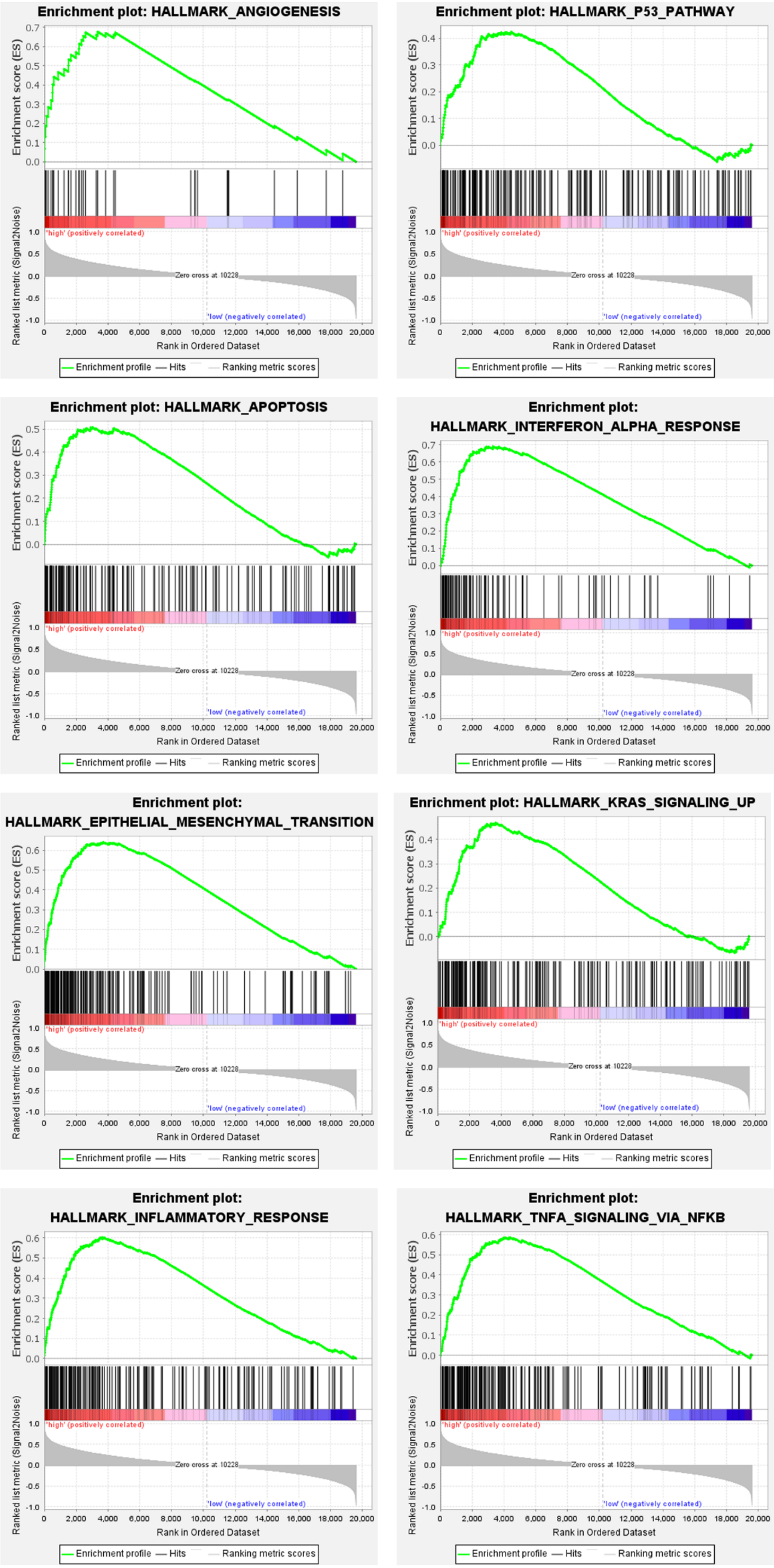

Enrichment plot: HALLMARK_ANGIOGENESIS
Enrichment plot: HALLMARK_P53_PATHWAY
Enrichment plot: HALLMARK_APOPTOSIS
Enrichment plot: HALLMARK_INTERFERON_ALPHA_RESPONSE
Enrichment plot: HALLMARK_EPITHELIAL_MESENCHYMAL_TRANSITION
Enrichment plot: HALLMARK_KRAS_SIGNALING_UP
Enrichment plot: HALLMARK_INFLAMMATORY_RESPONSE
Enrichment plot: HALLMARK_TNFA_SIGNALING_VIA_NFKB
Enrichment score (ES)
Ranked list metric (Signal2Noise)
'high' (positively correlated)
Zero cross at 10228
'low' (negatively correlated)
Rank in Ordered Dataset
Enrichment profile
Hits
Ranking metric scores

d

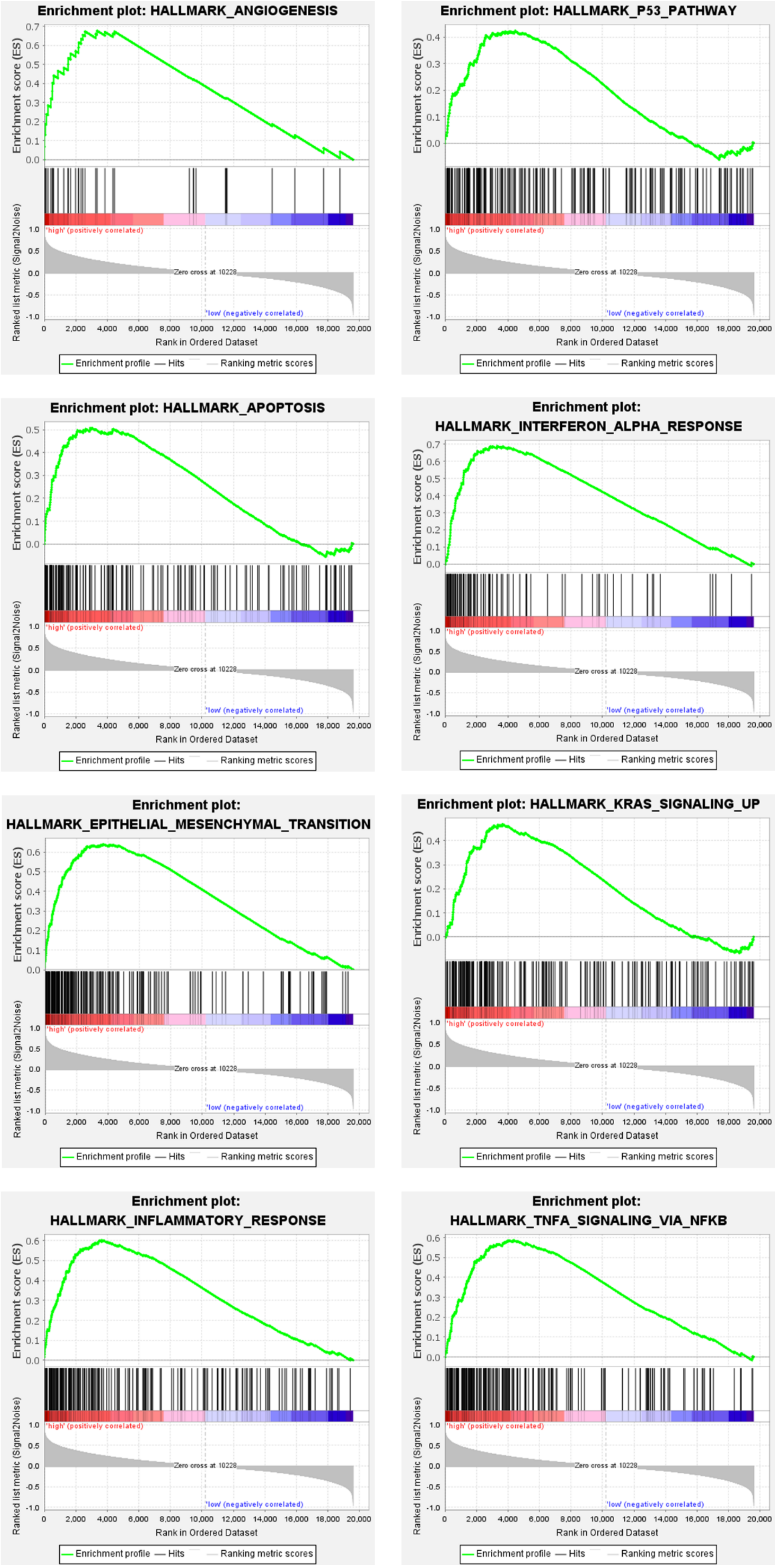
Enrichment plot: HALLMARK_ANGIOGENESIS
Enrichment plot: HALLMARK_P53_PATHWAY
Enrichment plot: HALLMARK_APOPTOSIS
Enrichment plot: HALLMARK_INTERFERON_ALPHA_RESPONSE
Enrichment plot: HALLMARK_EPITHELIAL_MESENCHYMAL_TRANSITION
Enrichment plot: HALLMARK_KRAS_SIGNALING_UP
Enrichment plot: HALLMARK_INFLAMMATORY_RESPONSE
Enrichment plot: HALLMARK_TNFA_SIGNALING_VIA_NFKB
Enrichment score (ES)
Ranked list metric (Signal2Noise)
'high' (positively correlated)
Zero cross at 10228
'low' (negatively correlated)
Rank in Ordered Dataset
Enrichment profile
Hits
Ranking metric scores

Supplementary Fig 3 GSEA analysis of data from the TCGA (**a**), CGGA (**b**), GSE16011 (**c**), and REMBRADANT (**d**) database showing the pathway enriched in the high-risk group. The nominal p value is less than 0.05, and the FDR q value is less than 0.25 in all figures. In each GSEA graph, the green curves in the upper panels represent the enrichment score (ES) of each gene in the ranking list; The black bars in the middle panels mean the position of each gene from a settled gene set (e.g. HALLMARK_ANGIOGENESIS) in the ranking gene list. The red gradients means the corresponding genes overexpress in high risk group while the blue gradients overexpress in the low risk group. The grey shading in the lower panels refer to Signal to Noise Ratio of each gene inputted into the GSEA software, and the genes aforementioned are ranked from highest to lowest.
